# Planned mode of birth after previous caesarean section and special educational needs in childhood: a population‐based record linkage cohort study

**DOI:** 10.1111/1471-0528.16828

**Published:** 2021-07-28

**Authors:** KE Fitzpatrick, JJ Kurinczuk, MA Quigley

**Affiliations:** ^1^ National Perinatal Epidemiology Unit Nuffield Department of Population Health University of Oxford Oxford UK

**Keywords:** Caesarean section, child outcomes, elective repeat caesarean section (ERCS), mode of birth, special educational needs, trial of labour after previous caesarean (TOLAC), vaginal birth after previous caesarean (VBAC)

## Abstract

**Objective:**

To investigate the association between planned mode of birth after previous caesarean section and a child’s risk of having a record of special educational needs (SENs).

**Design:**

Population‐based cohort study.

**Setting:**

Scotland.

**Population:**

A cohort of 44 892 singleton children born at term in Scotland between 2002 and 2011 to women with one or more previous caesarean sections.

**Methods:**

Linkage of Scottish national health and education data sets.

**Main outcome measures:**

Any SENs and specific types of SEN recorded when a child was aged 4–11 years and attending a Scottish primary or special school.

**Results:**

Children born following planned vaginal birth after previous caesarean (VBAC) compared with elective repeat caesarean section (ERCS) had a similar risk of having a record of any SENs (19.24 versus 17.63%, adjusted risk ratio aRR 1.04, 95% CI 0.99–1.09) or specific types of SEN. There was also little evidence that planned VBAC with or without labour induction compared with ERCS was associated with a child’s risk of having a record of any SENs (21.42 versus 17.63%, aRR 1.09, 95% CI 1.01–1.17 and 18.78 versus 17.63%, aRR 1.03, 95% CI 0.98–1.08, respectively) or most types of SEN. However, an increased risk of sensory impairment was seen for planned VBAC with labour induction compared with ERCS (1.18 versus 0.78%, risk difference 0.4%, adjusted odds ratio aOR 1.60, 95% CI 1.09–2.34).

**Conclusions:**

This study provides little evidence of an association between planned mode of birth after previous caesarean and SENs in childhood beyond a small absolute increased risk of sensory impairment seen for planned VBAC with labour induction. This finding may be the result of performing multiple comparisons or residual confounding. The findings provide valuable information to manage and counsel women with previous caesarean section concerning their future birth choices.

**Tweetable abstract:**

There is little evidence planned mode of birth after previous caesarean section is associated with special educational needs in childhood.

## Tweetable abstract

There is little evidence planned mode of birth after previous caesarean section is associated with special educational needs in childhood.

## Introduction

Globally, caesarean section rates are thought to have almost doubled between 2000 and 2015.^1^ In the UK, around 30% of all births now occur by caesarean section.^2^, ^3^, ^4^ There has been a corresponding increase in the number of pregnant women with a history of previous caesarean section. Policy in many high‐income settings supports offering such women a choice between planned elective repeat caesarean section (ERCS) or planned vaginal birth after previous caesarean (VBAC), in the absence of contraindications for VBAC. Although clinical guidelines recommend counselling women about the risks and benefits of this choice,^5^, ^6^, ^7^, ^8^ there remains a particular lack of evidence about the effect of this choice on the child’s longer‐term neurodevelopment.

Caesarean section may adversely affect a child’s neurodevelopment through several mechanisms, including altering the composition of the infant’s gut microbiota,^9^, ^10^, ^11^ affecting the stress response,^12^, ^13^ birth at an earlier gestation,^14^ or reducing the likelihood of breastfeeding.^15^, ^16^, ^17^, ^18^ However, although a recent meta‐analysis reported that both planned and emergency caesarean delivery were associated with an increased risk of autism spectrum disorder (ASD) and attention deficit/hyperactivity disorder, no significant association was evident for other neurodevelopmental and psychiatric outcomes examined, possibly reflecting the limited number of studies.^19^ Heterogeneity among studies was also high in the meta‐analysis of some of the outcomes assessed. Also, to our knowledge, to date only one study has examined the effect of planned mode of birth after previous caesarean on such outcomes.^20^ However, this study didn’t exclude children born to women with contraindications for planned VBAC and only investigated the effect on learning disability and cerebral palsy in around 8000 children. Research conducted in the general obstetric population or populations with specific complications, such as breech presentation,^21^, ^22^, ^23^, ^24^ may not be generalisable to the situation of birth after previous caesarean as it is more prone to the issue of confounding by indication, where adverse outcomes are related to the medical reasons that led to the caesarean section. Furthermore, several studies have demonstrated that planned VBAC is associated with an increased risk of serious birth‐related maternal and perinatal complications,^25^, ^26^, ^27^, ^28^ which may adversely affect a child’s longer‐term neurodevelopment. Therefore, planned VBAC may actually be associated with poorer child neurodevelopmental outcomes. This study aimed to investigate the association between planned mode of birth after previous caesarean section and a child’s risk of having a record of special educational needs (SENs), as a marker of neurodevelopmental adversity, among singleton children born at term to women considered clinically eligible to plan VBAC.

## Methods

A population‐based cohort study was conducted using Scottish national health and education (pupil census) data sets (for details of data sources, linkage methods, codes and database fields used, see Tables S1 and S2).

### Study population

**Figure 1:**
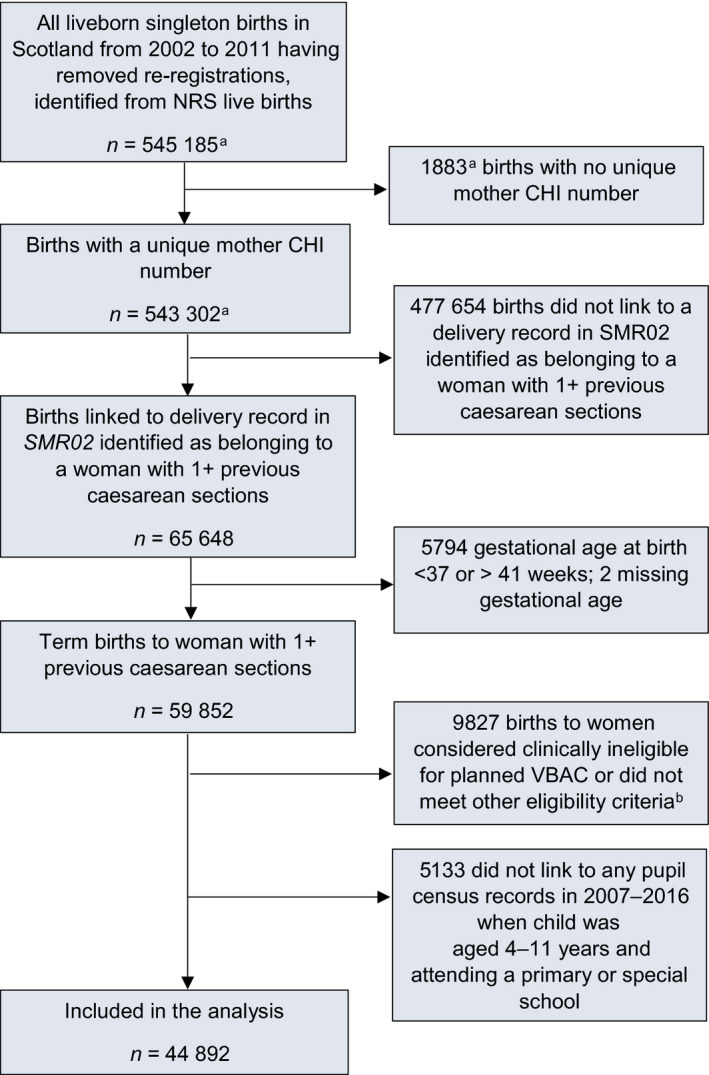
Flow diagram of cohort selection. ^a^Numbers provided by Information Services Division Scotland Scotland, now part of Public Health Scotland. ^b^Clinically ineligible for planned VBAC or did not meet other eligibility criteria for study because of one or more of the following: non‐cephalic presentation at birth (*n* = 4574); placenta praevia (*n* = 378); abdominal pregnancy (*n* = 0); known or suspected disproportion of maternal and/or fetal origin (*n* = 99); tumour of corpus uteri (*n* = 166); birth by prelabour non‐elective caesarean section (*n* = 4476); missing information on mode of birth (*n* = 9); birth by non‐elective caesarean section missing information about duration of labour (*n* = 507); number of previous caesarean sections greater than parity (*n* = 151); child died before the age of 4 years (*n* = 172). These reasons are not mutually exclusive. CHI, community health index; NRS, National Records of Scotland; *SMR02*, Scottish Morbidity Record Maternity Inpatient and Day Case data set; VBAC, vaginal birth after previous caesarean.

All live singleton term births (at 37–41 completed weeks of gestation) in Scotland, UK, between 1 January 2002 and 31 December 2011 to women with one or more previous caesarean sections were identified. Births to women not considered clinically eligible to plan VBAC based on current UK guidelines were excluded (Table S2),^5^, ^6^ in addition to births with missing or inconsistent data for key variables and children who died before the age of 4 years (Figure 1).

### Exposures

The main exposure was planned mode of birth after previous caesarean section, with planned VBAC (birth vaginally or by non‐elective caesarean with a duration of labour of ≥1 hour) compared with ERCS (birth by elective caesarean, defined by the Information Services Division Scotland as caesarean section performed during the day with both the patient and staff fully prepared). Additional analyses compared planned VBAC with or without labour induction and ERCS. Analyses were also performed by actual mode of birth after previous caesarean section, comparing children born by VBAC (birth vaginally), those born by in‐labour non‐elective repeat caesarean section (birth by non‐elective caesarean with a duration of labour of ≥1 hour) and those born by ERCS.

### Outcomes

The primary outcome was any record of SENs and the secondary outcomes were the specific type of SEN, with children classified as having a record of SENs if it was recorded in any pupil census year when they were aged 4–11 years and attending a Scottish primary or special school (school providing education to children with complex or specific needs that cannot be met in mainstream schools). The Education (Additional Support for Learning) Scotland Act 2004 (as amended) defines SENs as being unable to benefit from the school education provided without the provision of additional support beyond that normally given to schoolchildren of the same age. Educational authorities have a legal duty to identify and provide support for children with SENs. For this study, SENs arising from the following causes were included: learning disability; dyslexia; other specific or moderate learning difficulty; sensory impairment; physical or motor impairment; language or speech disorder; ASD; social, emotional and behavioural difficulty or mental health problem; and physical health problem. A child can be recorded as having more than one type of SEN.

### Statistical analysis

All analyses were prespecified as described in the methods. Associations between the exposures and outcomes were examined using logistic regression to estimate odds ratios (ORs), where the outcome was rare (affecting <10% of population), and modified Poisson regression to estimate risk ratios (RRs),^29^ where the outcome was common, recognising that ORs do not give a good approximation of RRs when the outcome is common.^30^ To account for temporal changes, all models were adjusted for year of birth. Models were then sequentially adjusted for covariates determined a priori based on pre‐existing hypotheses or evidence on what factors are believed to potentially explain any associations between the exposure and the outcome in question,^14^, ^22^, ^25^, ^31^, ^32^ with a conceptual framework of how the factors might influence the relationships investigated outlined in Figure S1. Model A adjusted for sociodemographic, maternal medical and pregnancy‐related factors (detailed in Table 1), model B additionally adjusted for infant‐related factors (detailed in Table 1) and model C  additionally adjusted for whether the mother breastfed (exclusively or mixed) the infant around 6–8 weeks postpartum to examine any effects of the exposures that were not mediated through breastfeeding, recognising that breastfeeding might be on the causal pathway.^16^, ^17^, ^18^, ^27^ Evidence of effect modification between the exposures and any breastfeeding at 6–8 weeks postpartum was examined, in addition to investigating evidence of effect modification between the main exposure and the following covariates: number of previous caesarean sections, any prior vaginal birth and gestational age at birth. Effect modification was explored by adding interaction terms to the full models. The linearity of continuous variables was investigated using fractional polynomials. Where there was evidence in the complete case analysis of effect modification or nonlinear covariate effects, an extension of the multiple imputation by chained equations method was used to impute partially observed covariates.^33^ Otherwise, the normal multiple imputation by chained equations approach was used, including all covariates and the outcome of interest and performing 55 imputations. To account for correlations between observations relating to more than one eligible child born to the same mother, robust standard errors clustered on the mother ID were used.

**Table 1:** Characteristics of study cohort by planned mode of birth after previous caesarean section

	ERCS No. (%)^a^, unless otherwise stated (*n* = 26 041)	Planned VBAC No. (%)^a^, unless otherwise stated (*n* = 18 851)	*P*
Sociodemographic, maternal medical and pregnancy‐related characteristics			
Maternal age (years)			
<25	2706 (10.4)	2501 (13.3)	
25–29	5673 (21.8)	4336 (23.0)	
30–34	8962 (34.4)	6573 (34.9)	
35–39	7073 (27.2)	4555 (24.2)	
40 or more	1627 (6.2)	886 (4.7)	<0.001
Median (IQR) maternal age (years)	32 (28–36)	32 (27–35)	<0.001
Mother’s country of birth			
UK	24 050 (92.4)	17 144 (90.9)	
Non‐UK	1991 (7.6)	1707 (9.1)	<0.001
Marital status/registration type			
Married or joint registration/same address	23 478 (90.2)	16 696 (88.6)	
Joint registration/different address	1688 (6.5)	1415 (7.5)	
Sole registration	875 (3.4)	740 (3.9)	<0.001
Socioeconomic status^b^			
Managerial/professional	12 184 (46.8)	8388 (44.5)	
Intermediate	6072 (23.3)	4158 (22.1)	
Routine/manual	6844 (26.3)	5424 (28.8)	
Other^c^	941 (3.6)	881 (4.7)	<0.001
Child’s ethnicity^d^			
White	24 205 (94.9)	17 176 (92.7)	
Asian	777 (3.0)	882 (4.8)	
African/Caribbean/Back/Mixed/Other	528 (2.1)	476 (2.6)	<0.001
Number of previous caesarean sections			
1	19 284 (74.1)	18 261 (96.9)	
2 or more	6757 (25.9)	590 (3.1)	<0.001
Median (IQR) number of previous caesarean sections	1 (1–2)	1 (1–1)	<0.001
Any prior vaginal birth^d^			
No	21 541 (83.2)	11 622 (61.9)	
Yes	4365 (16.8)	7160 (38.1)	<0.001
Parity^d^			
1	16 125 (62.3)	11 338 (60.5)	
2 or more	9767 (37.7)	7397 (39.5)	<0.001
Median (IQR) parity^d^	1 (1–2)	1 (1–2)	<0.001
Interpregnancy interval (months)^d^			
24 or more	14 796 (60.4)	9468 (56.8)	
12–23	6273 (25.6)	4495 (27.0)	
<12	3444 (14.0)	2708 (16.2)	<0.001
Median (IQR) interpregnancy interval (months)^d^	29.5 (17.0–49.9)	27.7 (15.8–48.8)	<0.001
Mother smoked at booking^d^			
No	19 168 (81.7)	13 114 (76.1)	
Yes	4303 (18.3)	4124 (23.9)	<0.001
Maternal BMI at booking (kg/m^2^)^d^			
<25	5623 (35.3)	4766 (45.4)	
25–29.9	4925 (30.9)	3294 (31.4)	
30 or more	5375 (33.8)	2433 (23.2)	<0.001
Median (IQR) BMI at booking (kg/m^2^)^d^	27.1 (23.7–32.1)	25.5 (22.7–29.4)	<0.001
Any hypertensive disorder	1600 (6.1)	1219 (6.5)	0.165
Pre‐existing or gestational diabetes	865 (3.3)	231 (1.2)	<0.001
Prelabour rupture of membranes	263 (1.0)	1488 (7.9)	<0.001
Infant‐related characteristics			
Male infant	13 169 (50.6)	9720 (51.6)	0.038
Gestational age at birth (weeks)			
39–41	17 110 (65.7)	15 179 (80.5)	
37–38	8931 (34.3)	3672 (19.5)	<0.001
Median (IQR) gestational age at birth (weeks)	39 (38–39)	40 (39–40)	<0.001
Birthweight centile^d^			
10‐90th	20 298 (78.4)	15 117 (80.4)	
Less than 10th	1555 (6.0)	1992 (10.6)	
More than 90th	4021 (15.5)	1688 (9.0)	<0.001
Other characteristics			
Adverse perinatal outcome^d^, ^e^			
No	20 783 (93.4)	15 262 (91.7)	
Yes	1470 (6.6)	1384 (8.3)	<0.001
Maternal intrapartum or postpartum complication^f^			
No	25 250 (97.0)	17 606 (93.4)	
Yes	791 (3.0)	1245 (6.6)	<0.001
Any breastfeeding at 6–8 week review^d^			
No	14 586 (67.3)	9201 (59.4)	
Yes	7102 (32.7)	6279 (40.6)	<0.001

BMI, body mass index; ERCS, elective repeat caesarean section; IQR, interquartile range; NS‐SEC, National Statistics Socio‐Economic Classification; VBAC, vaginal birth after previous caesarean.

a Percentage of those with complete data.

b Socioeconomic status of mother for sole registered birth or highest between mother’s and father’s socioeconomic status for births registered inside marriage or jointly registered by both parents outside marriage. Socioeconomic status defined by NS‐SEC based on occupation and employment status.

c Other includes never worked/long‐term unemployed, student, not stated or not classifiable.

d Missing data: child’s ethnicity, 848 (1.89%); any prior vaginal birth, 204 (0.45%); parity, 265 (0.59%); interpregnancy interval, 3708 (8.26%); maternal smoking status, 4183 (9.32%); maternal BMI, 18 476 (41.16%); birthweight centile, 221 (0.49%); adverse perinatal outcome, 5993 (13.35%); any breastfeeding at 6–8 weeks postpartum, 7724 (17.21%). Overall, 55.6% of the study population had missing data on one or more of the covariates included in the fully adjusted models.

e Adverse perinatal outcome includes admission to a neonatal unit, resuscitation with drugs and/or intubation or an Apgar score <7 at 5 minutes.

f Intrapartum or postpartum complication includes uterine rupture, peripartum hysterectomy, blood transfusion, puerperal sepsis, other puerperal infection, surgical injury (damage to bowel, bladder or ureter requiring surgical repair) or third‐ or fourth‐degree perineal tear.

Several sensitivity analyses were conducted. First, complete case analyses were performed. Second, as the criterion used to identify planned mode of birth could misclassify women who planned ERCS but went into labour before their scheduled caesarean date, analyses were repeated, limiting to the gestation recommended by UK guidelines to perform an ERCS (≥39 weeks of gestation).^5^, ^6^ Third, SENs were analysed as a repeated‐measures yearly outcome using robust standard errors clustered on child ID to account for correlations between observations relating to the same child across different school years. As this approach can only account for one source of non‐independence, a woman’s first eligible child was selected where there was more than one eligible child born to the same mother. For this sensitivity analysis the age of the child at the time of the pupil census was included as a covariate in all models, and evidence of effect modification between the main exposure and the age of the child was assessed. Fourth, given the high proportion of missing data for the covariate maternal body mass index (BMI) at booking for pregnancy care, analyses were repeated omitting this covariate. All analyses were conducted in Stata mp  version 16.

### Patient and public involvement

As part of a larger programme of research, this study was supported by patient and public involvement (PPI) representatives from various service user and voluntary groups in the perinatal field. PPI representatives provided input on the proposed research plan, including the research questions, when developing the funding application for the programme of research. PPI representatives have also commented on the findings so far and have contributed to the dissemination plan for the programme of research. This will include producing, with the help of the PPI representatives, a plain language summary leaflet of the research findings.

## Results

In total, 50 025 live singleton term births to women with one or more previous caesarean sections met the eligibility criteria for the study. Of these, 5133 (10.3%) were excluded because they did not link to any pupil census records between 2007 and 2016, when the child was aged 4–11 years and attending a primary of special school (Figure 1). The characteristics of the 44 892 children included in the analysis, compared with the 5133 excluded, are shown in Table S3. The mothers of the children not included were more likely to be older than the mothers of the children included, born outside of the UK, be married or cohabitating, be of higher socioeconomic status, have a shorter inter‐pregnancy interval and breastfed at 6‐8 weeks postpartum. They were less likely than the mothers of the children included to be smokers at booking for pregnancy care, obese or have a hypertensive disorder, whereas the other characteristics including planned mode of birth were comparable.

Of the children included, 18 851 (42.0%) were born following planned VBAC and 26 041 (58.0%) were born by ERCS. The ERCS rate increased from 50.2% in 2002 to 64.7% in 2011. The mothers of children born following planned VBAC were more likely than those born by ERCS to be younger, born outside the UK and have a lower socioeconomic status (Table 1). They were also more likely to have just one prior caesarean section, have had one or more prior vaginal births, a shorter interpregnancy interval, be smokers at booking for pregnancy care, have prelabour rupture of membranes, experienced an adverse perinatal outcome or maternal intrapartum or postpartum complication and have breastfed at 6–8 weeks postpartum. They were less likely to be married or cohabitating, to be obese or to have diabetes. Children born following planned VBAC were less likely than those born by ERCS to be of white ethnicity, female, born at early term (37–38 weeks of gestation) and have a larger birthweight for their gestational age at birth.

Overall, 18.3% of the children included in the analysis had any record of SENs when they were aged 4–11 years and attending a primary or special school: 2.9% had a learning disability; 2.1% had dyslexia; 8.3% had other specific or moderate learning difficulties; 0.8% had sensory impairment; 1.0% had physical or motor impairment; 3.8% had a language or speech disorder; 1.6% had ASD; 4.8% had a social, emotional and behavioural difficulty or mental health problem; and 1.5% had a physical health problem. Moreover, 5.7% of children had more than one type of SEN. Outcomes by planned mode of birth are reported in Table 2 and Figure S2A. In the fully adjusted models, compared with ERCS planned VBAC was associated with a similar risk of the child having a record of any SENs or a specific type of SEN.

**Table 2:** Outcomes following planned VBAC, compared with ERCS

Outcomes	ERCS No. (%) outcome events (*n* = 26 041)	Planned VBACNo. (%) outcome events (*n* = 18 851)	Base model^a^OR or RR (95% CI)	Model A^b^OR or RR (95% CI)	Model B^c^OR or RR (95% CI)	Model C^d^OR or RR (95% CI)
Any SENs	4592 (17.63)	3627 (19.24)	1.03 (0.99–1.07)	1.02 (0.98–1.07)	1.04 (0.99–1.08)	1.04 (0.99–1.09)
			*P* = 0.194	*P* = 0.363	*P* = 0.134	*P* = 0.099
Learning disability	712 (2.73)	581 (3.08)	1.02 (0.91–1.14)	1.02 (0.90–1.16)	1.06 (0.93–1.22)	1.08 (0.94–1.24)
			*P* = 0.754	*P* = 0.759	*P* = 0.392	*P* = 0.292
Dyslexia	491 (1.89)	440 (2.33)	1.02 (0.89–1.17)	1.11 (0.95–1.29)	1.11 (0.95–1.30)	1.09 (0.94–1.28)
			*P* = 0.765	*P* = 0.184	*P* = 0.191	*P* = 0.260
Other specific or moderate learning difficulty	2064 (7.93)	1644 (8.72)	1.02 (0.95–1.09)	1.02 (0.94–1.10)	1.04 (0.96–1.13)	1.05 (0.97–1.14)
			*P* = 0.658	*P* = 0.640	*P* = 0.311	*P* = 0.251
Sensory impairment	204 (0.78)	165 (0.88)	1.05 (0.85–1.30)	1.10 (0.87–1.40)	1.18 (0.92–1.52)	1.17 (0.91–1.50)
			*P* = 0.633	*P* = 0.430	*P* = 0.190	*P* = 0.220
Physical or motor impairment	259 (0.99)	204 (1.08)	1.03 (0.86–1.24)	1.08 (0.87–1.34)	1.14 (0.91–1.43)	1.15 (0.92–1.44)
			*P* = 0.731	*P* = 0.485	*P* = 0.240	*P* = 0.226
Language or speech disorder	983 (3.77)	719 (3.81)	1.02 (0.92–1.12)	1.03 (0.92–1.15)	1.01 (0.90–1.13)	1.02 (0.91–1.15)
			*P* = 0.762	*P* = 0.646	*P* = 0.891	*P* = 0.757
Autistic spectrum disorder	419 (1.61)	302 (1.60)	0.97 (0.84–1.13)	1.00 (0.84–1.18)	1.03 (0.86–1.22)	1.02 (0.86–1.22)
			*P* = 0.732	*P* = 0.963	*P* = 0.780	*P* = 0.803
Social, emotional & behavioural difficulty or mental health problem	1162 (4.46)	997 (5.29)	**1.12 (1.02–1.22)**	1.03 (0.93–1.14)	1.05 (0.94–1.17)	1.06 (0.95–1.18)
			***P* =** **0.017**	*P* = 0.598	*P* = 0.359	*P* = 0.289
Physical health problem	381 (1.46)	285 (1.51)	0.98 (0.84–1.15)	1.05 (0.88–1.25)	1.07 (0.89–1.29)	1.08 (0.90–1.30)
			*P* = 0.822	*P* = 0.617	*P* = 0.449	*P* = 0.414

BMI, body mass index; CI, confidence interval; ERCS, elective repeat caesarean section; OR, odds ratio; RR, risk ratio; SENs, special educational needs; VBAC, vaginal birth after previous caesarean.

Bold text indicates statistically significant findings at the 5% level.

a Base model adjusted for year of birth only.

b Model A adjusted for year of birth, sociodemographic (maternal age, mother’s country of birth, marital status, socioeconomic status and child’s ethnicity) and maternal medical and pregnancy‐related factors (number of previous caesarean sections, any prior vaginal birth, inter‐pregnancy interval, maternal smoking status at booking, maternal BMI at booking, hypertensive disorder, diabetes and prelabour rupture of membranes).

c Model B adjusted for variables in model A and additionally adjusted for infant‐related factors (sex of infant, gestational age at birth and birthweight centile).

d Model C adjusted for variables in model B and additionally adjusted for any breastfeeding at 6–8 weeks postpartum.

Of the children born following planned VBAC, 17.6% (3305/18 793) were born to mothers who had their labour induced (25.8% using only surgical methods, 26.9% using only medical methods and 46.6% using surgical and medical methods). Outcomes following planned VBAC with or without labour induction, compared with ERCS, are presented in Table 3 and Figure S2C and S2B, respectively. In the fully adjusted models, compared with ERCS, a planned VBAC with labour induction was associated with only a slight increase in the risk of any SENs (RR 1.09, 95% CI 1.01–1.17), but a larger increase in risk was evident for sensory impairment (OR 1.60, 95% CI 1.09–2.34), although the absolute risk difference for sensory impairment was small (0.4%). No other significant differences in outcomes were evident following VBAC with or without labour induction, compared with ERCS, in the fully adjusted models.

**Table 3:** Outcomes following planned VBAC with and without labour induction, compared with ERCS

Outcomes	ERCS	Planned VBAC without labour induction					Planned VBAC with labour induction				
	No. (%) outcome events (*n* = 26 041)	No. (%) outcome events (*n* = 15 488)	Base model^a^OR or RR(95% CI)	Model A^b^OR or RR(95% CI)	Model B^c^OR or RR(95% CI)	Mode C^d^OR or RR(95% CI)	No. (%) outcome events (*n* = 3305)	Base model^a^OR or RR(95% CI)	Model A^b^OR or RR(95% CI)	Model B^c^OR or RR(95% CI)	Model C^d^OR or RR(95% CI)
Any SENs	4592 (17.63)	2909 (18.78)	1.01 (0.96–1.05)	1.01 (0.97–1.06)	1.03 (0.98–1.08)	1.03 (0.98–1.08)	708 (21.42)	**1.13**	1.05	**1.08**	**1.09**
								**(1.05–1.21)**	(0.98–1.13)	**(1.01–1.17)**	**(1.01–1.17)**
			*P = *0.767	*P = *0.535	*P = *0.274	*P = *0.212		***P =*** * * **0.001**	*P = *0.201	***P =*** * * **0.033**	***P =*** * * **0.027**
Learning disability	712 (2.73)	467 (3.02)	1.01 (0.89–1.14)	1.02 (0.89–1.17)	1.06 (0.92–1.22)	1.07 (0.93–1.24)	114 (3.45)	1.09	1.02	1.11	1.12
								(0.89–1.33)	(0.82–1.26)	(0.88–1.39)	(0.89–1.40)
			*P = *0.910	*P = *0.728	*P = *0.439	*P = *0.331		*P = *0.432	*P = *0.854	*P = *0.389	*P = *0.338
Dyslexia	491 (1.89)	346 (2.23)	1.00 (0.86–1.15)	1.08 (0.92–1.27)	1.09 (0.93–1.28)	1.07 (0.91–1.26)	92 (2.78)	1.11	1.20	1.21	1.20
								(0.88–1.40)	(0.94–1.54)	(0.93–1.57)	(0.92–1.55)
			*P = *0.955	*P = *0.327	*P = *0.300	*P = *0.395		*P = *0.361	*P = *0.144	*P = *0.148	*P = *0.174
Other specific or moderate learning difficulty	2064 (7.93)	1321 (8.53)	1.00 (0.92–1.07)	1.02 (0.94–1.10)	1.04 (0.95–1.13)	1.04 (0.96–1.14)	319 (9.65)	1.11	1.04	1.09	1.09
								(0.98–1.27)	(0.91–1.19)	(0.95–1.25)	(0.95–1.26)
			*P = *0.928	*P = *0.710	*P = *0.402	*P = *0.329		*P = *0.097	*P = *0.575	*P = *0.227	*P = *0.203
Sensory impairment	204 (0.78)	124 (0.80)	0.97 (0.77–1.22)	1.02 (0.79–1.33)	1.09 (0.84–1.42)	1.08 (0.83–1.41)	39 (1.18)	1.38	1.43	**1.61**	**1.60**
								(0.97–1.97)	(0.99–2.07)	**(1.10–2.36)**	**(1.09–2.34)**
			*P = *0.794	*P = *0.869	*P = *0.527	*P = *0.583		*P = *0.072	*P = *0.060	***P =*** * * **0.015**	***P =*** * * **0.017**
Physical or motor impairment	259 (0.99)	167 (1.08)	1.03 (0.85–1.26)	1.09 (0.87–1.36)	1.14 (0.91–1.44)	1.15 (0.91–1.45)	36 (1.09)	1.01	1.02	1.12	1.12
								(0.71–1.44)	(0.71–1.46)	(0.76–1.64)	(0.77–1.65)
			*P = *0.734	*P = *0.462	*P = *0.257	*P = *0.242		*P = *0.947	*P = *0.936	*P = *0.561	*P = *0.549
Language or speech disorder	983 (3.77)	588 (3.80)	1.01	1.03 (0.92–1.16)	1.01 (0.90–1.14)	1.02 (0.91–1.16)	129 (3.90)	1.06	1.01	1.00	1.00
			(0.90–1.12)					(0.88–1.28)	(0.83–1.22)	(0.81–1.22)	(0.81–1.23)
			*P = *0.895	*P = *0.597	*P = *0.836	*P = *0.703		*P = *0.528	*P = *0.956	*P = *0.942	*P = *0.994
Autistic spectrum disorder	419 (1.61)	241 (1.56)	0.95 (0.81–1.11)	0.98 (0.82–1.18)	1.01 (0.84–1.21)	1.00 (0.83–1.21)	60 (1.82)	1.09	1.05	1.12	1.12
								(0.83–1.45)	(0.78–1.41)	(0.83–1.52)	(0.83–1.51)
			*P = *0.519	*P = *0.858	*P = *0.953	*P = *0.978		*P = *0.522	*P = *0.744	*P = *0.455	*P = *0.465
Social, emotional and behavioural difficulty or mental health problem	1162 (4.46)	796 (5.14)	1.09 (0.99–1.20)	1.03 (0.92–1.14)	1.04 (0.93–1.17)	1.05 (0.94–1.18)	201 (6.08)	**1.26**	1.05	1.11	1.12
								**(1.08–1.48)**	(0.89–1.24)	(0.93–1.33)	(0.94–1.34)
			*P = *0.078	*P = *0.629	*P = *0.448	*P = *0.369		***P =*** * * **0.004**	*P = *0.547	*P = *0.229	*P = *0.199
Physical health problem	381 (1.46)	225 (1.45)	0.95 (0.80–1.12)	1.02 (0.85–1.23)	1.04 (0.86–1.26)	1.05 (0.87–1.27)	60 (1.82)	1.16	1.19	1.26	1.26
								(0.88–1.53)	(0.89–1.58)	(0.93–1.70)	(0.93–1.71)
			*P = *0.526	*P = *0.832	*P = *0.656	*P = *0.614		*P = *0.290	*P = *0.250	*P = *0.137	*P = *0.130

BMI, body mass index; CI, confidence interval; ERCS, elective repeat caesarean section; OR, odds ratio; RR, risk ratio; SENs, special educational needs; VBAC, vaginal birth after previous caesarean.

Bold text indicates statistically significant findings at the 5% level.

a Base model adjusted for year of birth only.

b Model A adjusted for year of birth, sociodemographic (maternal age, mother's country of birth, marital status, socioeconomic status and child’s ethnicity) and maternal medical and pregnancy‐related factors (number of previous caesarean sections, any prior vaginal birth, inter‐pregnancy interval, maternal smoking status at booking, maternal BMI at booking, hypertensive disorder, diabetes and prelabour rupture of membranes).

c Model B, adjusted for variables in model A and additionally adjusted for infant‐related factors (sex of infant, gestational age at birth and birthweight centile).

d Model C, adjusted for variables in model B and additionally adjusted for any breastfeeding at 6–8 weeks postpartum.

Of the children born following planned VBAC, 72.0% (13 564/18 851) were actually born by VBAC and 28.0% (5287/18 851) were born by in‐labour non‐elective repeat caesarean section. There was no strong evidence that actual mode of birth after previous caesarean section was associated with a child’s risk of having a record of any SENs or most types of SEN (Figure S2D and S2E; Table S4). In the fully adjusted models, only a slight increase in the risk of any SENs (RR 1.06, 95% CI 1.01–1.12), other specific or moderate learning difficulties (OR 1.10, 95% CI 1.00–1.20) and a language or speech disorder (OR 1.14, 95% CI 1.00–1.30) was evident with actual VBAC compared with ERCS. By contrast, a reduced risk of language or speech disorder was seen with in‐labour non‐elective repeat caesarean, compared with ERCS (fully adjusted OR 0.76, 95% CI 0.63–0.92), although the absolute risk difference was small (0.99%)

There was no evidence of effect modification by breastfeeding status at 6–8 weeks postpartum, the number of prior caesarean sections that the mother had, whether the mother had any prior vaginal birth, gestational age at birth or, in the repeated‐measures sensitivity analysis, the age of the child. The sensitivity analyses (Tables S5–S16) largely yielded similar effect estimates, which were at least in the range of those reported for the main analysis, although the statistical significance of some associations varied, noting that the sensitivity analyses included a smaller number of children.

## Discussion

### Main findings

In this study of 44 892 children born to women considered clinically eligible to plan VBAC, planned VBAC compared with ERCS was associated with a similar risk of the child having a record of any SENs or a specific type of SEN when they were aged 4–11 years and attending a primary or special school. However, compared with ERCS, planned VBAC with labour induction was associated with a 60% increased risk of sensory impairment, although the absolute risk difference was small (0.4%). There was also evidence of a 24% reduced risk of language or speech disorder with in‐labour non‐elective repeat caesarean, compared with ERCS, although the absolute risk difference was again small (0.99%). As discussed subsequently, these differences may be the result of performing multiple comparisons or residual confounding.

### Strengths and limitations

The study strengths include its large population‐based design, the use of national prospectively collected data subject to regular quality checks, restriction to children born to women considered clinically eligible to plan VBAC and the ability to explore the influence of many a priori covariates. However, the possibility of residual confounding remains. Although a large randomised controlled trial could overcome this limitation, a previous study suggested that such a trial is unlikely to be feasible.^28^ Therefore, large population‐based observational studies, like ours, provide the best opportunity to inform evidence in this area.

Our study only included children who could be linked to pupil census records, with likely explanations for any gaps in linking including that the child had emigrated from Scotland or had attended private school (around 4% of children in Scotland attend private schools and are not included in the pupil census).^34^ However, this is unlikely to have significantly biased our findings given that only 10.3% of children could not be linked,^35^ and the planned mode of birth in those who could and could not be linked was similar. Although we acknowledge that the criteria used to identify planned mode of birth may have misclassified women who planned ERCS but went into labour before their scheduled caesarean date, limiting the analysis to births at the gestation recommended by UK guidelines to perform an ERCS did not fundamentally change the findings. We also recognise that the proportion of children recorded with SENs in Scotland has increased over the study period, probably through changes and improvements in the recording of SENs.^36^ However, all our analyses were adjusted for year of birth so this should not have affected the reported relative effect estimates. The overall proportion of children in our study population identified with any SENs (18.3%) is in line with estimates using aggregate SEN data from the pupil census years 2007–2016, provided by the Scottish Government Educational Analytical Services, and figures in published summary reports based on pupil census data during the study period.^37^ Our results are likely to be generalisable to settings with similar clinical practice and school support, such as in England, where the overall prevalence of SENs over the study period ranged from around 14 to 21% and the most common primary type of SEN was moderate learning difficulty, comparable with our study findings.^38^ Missing covariate data, particularly for maternal BMI, is acknowledged as another limitation. However, our use of multiple imputation is considered an appropriate method for overcoming this, providing the unobserved data are missing at random and the imputation models have been specified correctly.^39^ Furthermore, the sensitivity analysis omitting maternal BMI from the models had little material impact on the effect estimates. Finally, although all analyses were prespecified based on clear hypotheses and biological plausibility, it is acknowledged that the performance of multiple comparisons would have increased the risk of type‐1 error.

### Interpretation

To our knowledge, to date only one study has investigated the effect of planned VBAC compared with ERCS on child neurodevelopmental outcomes.^20^ Consistent with our findings, this prior study reported no significant differences in the risk of learning disability or cerebral palsy among around 8000 singleton school‐aged children in two Scottish health boards born at term following planned VBAC, compared with ERCS. However, this prior study did find an increased risk of learning disability with non‐elective repeat caesarean compared with actual VBAC, which our findings do not suggest. This might reflect that the prior study, unlike our study, included prelabour non‐elective repeat caesarean sections, which are potentially more prone to confounding by indication. No other types of SENs were investigated in the prior Scottish study. Another study of around 1200–3500 Australian children, depending on the outcome considered, reported no differences in child development at the age of 5 years and in school achievement at the age of 8 years between children born by actual VBAC compared with ERCS.^40^ Compared with ERCS, our study only found a slight increase in the risk of any SENs and in certain types of SEN with actual VBAC, which might well be attributable to type‐1 error or residual confounding. As we are not aware of any clinical reason to explain the reduced risk of language or speech disorder seen only with in‐labour non‐elective repeat caesarean, compared with ERCS, this finding might also be attributable to type‐1 error or residual confounding.

It is possible that the slight increase in risk of any SENs and the increased risk of sensory impairment seen for planned VBAC with labour induction may be related to the increased risk of complications, most notably uterine rupture, that has been observed, particularly with inducing labour in women with previous caesarean section.^26^, ^41^ The increased risks seen may alternatively be related to the underlying indications for induction or the induction agents themselves, with concerns raised, for example, that excess circulating oxytocin may reach the neonate’s brain and desensitize the oxytocin receptors, leading to adverse developmental effects.^42^ However, with any of these potential explanations, one might expect to see an increased risk for other types of SEN with planned VBAC with labour induction, which was not the case. The increased risks that were seen with planned VBAC with labour induction may also therefore be the result of performing multiple comparisons or residual confounding. To our knowledge, our study is the first to have examined the effect of labour induction after previous caesarean on child neurodevelopmental outcomes. The small number of previous studies that have investigated the effect of labour induction on such outcomes in the general obstetric population have reported mixed findings.^43^

## Conclusion

This study provides little evidence of an association between planned mode of birth after previous caesarean section and SENs beyond a small absolute increased risk of sensory impairment seen for planned VBAC with labour induction. This finding may be the result of performing multiple comparisons or residual confounding. The findings provide valuable additional information to manage and counsel women with previous caesarean section concerning their future birth choices, but further research is needed to establish whether the results can be replicated and to explore the impact of planned mode of birth after previous caesarean section on other measures of child neurodevelopmental adversity.

### Disclosure of interests

KEF received financial support from the National Institute for Health Research (NIHR) for the submitted work. Completed disclosure of interests form available to view online as supporting information. The other authors reported none.

### Contribution to authorship

KEF conceived the study, gained funding and approvals for the study, cleaned and analysed the data and wrote the first draft of the article. MAQ and JJK supervised the study. All authors contributed to the design and interpretation of the study and critically reviewed the article and approved the final version for publication.

### Details of ethics approval

The study was exempt from UK National Research Ethics Service approval as it involved secondary analysis of anonymised data. However, study approval was obtained from the Public Benefit and Privacy Panel for Health and Social Care Scotland (1516‐0196) and the Scottish Government Education Analytical Services.

### Funding

KEF was funded by a National Institute for Health Research (NIHR) Doctoral Research Fellowship (DRF‐2016‐09‐078) for this research project. This paper presents independent research. The views expressed are those of the author(s) and not necessarily those of the NHS, the NIHR or the Department of Health and Social Care. The funders had no role in study design, data collection and analysis, decision to publish or preparation of the article.

### Acknowledgements

The authors would like to acknowledge the support of the eDRIS Team (National Services Scotland) for their involvement in obtaining approvals and provisioning and linking data, and the use of the secure analytical platform within the National Safe Haven.

### Data availability statement

Data (eDRIS study number 1516‐0196) are available from the Information Services Division Scotland, now part of Public Health Scotland, following the necessary approval process by the Public Benefit and Privacy Panel for Health and Social Care and the Scottish Government Education Analytical Services. More details can be found at https://www.isdscotland.org/Products‐and‐Services/EDRIS/ and at https://www.gov.scot/publications/scottish‐government‐statistics‐request‐our‐data/.

## Supporting information


**Figure S1**. Conception framework of how sociodemographic, maternal medical and pregnancy‐related and infant‐related factors might influence the relationship between planned mode of birth after previous caesarean and special educational needs (SENs) in the child.
**Figure S2**. Outcomes following: (A) planned VBAC, compared with ERCS; (B) planned VBAC without labour induction, compared with ERCS; (C) planned VBAC with labour induction, compared with ERCS; (D) planned and actual VBAC, compared with ERCS; and (E) planned VBAC but in‐labour non‐elective repeat caesarean section, compared with ERCS.
**Table S1**. Data sources.
**Table S2**. Data sources, codes and database fields used to identify study population, exposures, outcomes and covariates.
**Table S3**. Characteristics of children included in the study compared with those not included because they did not link to any pupil census records between 2007 and 2016 when the child was aged 4–11 years and attending a primary or special school.
**Table S4**. Outcomes according to actual mode of birth: planned and actually had VBAC and planned VBAC but had in‐labour non‐elective repeat caesarean section, compared with ERCS.
**Table S5**. Complete case analysis of outcomes following planned VBAC, compared with ERCS.
**Table S6**. Complete case analysis of outcomes following planned VBAC with and without labour induction, compared with ERCS.
**Table S7**. Complete case analysis of outcomes according to actual mode of birth: planned and actual VBAC and planned VBAC but in‐labour non‐elective repeat caesarean section, compared with ERCS.
**Table S8**. Outcomes following planned VBAC compared with ERCS at ≥ 39 weeks of gestation.
**Table S9**. Outcomes following planned VBAC with and without labour induction, compared with ERCS, at ≥39 weeks of gestation.
**Table S10**. Outcomes according to actual mode of birth: planned and actual VBAC and planned VBAC but in‐labour non‐elective repeat caesarean section, compared with ERCS, at ≥39 weeks of gestation.
**Table S11**. Outcomes following planned VBAC compared with ERCS, analysing SENs as a repeated‐measures yearly outcome.
**Table S12**. Outcomes following planned VBAC with and without labour induction compared with ERCS, analysing SENs as a repeated‐measures yearly outcome.
**Table S13**. Outcomes according to actual mode of birth: planned and actual VBAC and planned VBAC but in‐labour non‐elective repeat caesarean section, compared with ERCS, analysing SENs as a repeated‐measures yearly outcome.
**Table S14**. Outcomes following planned VBAC compared with ERCS, removing covariate maternal BMI from the analysis.
**Table S15**. Outcomes following planned VBAC with and without labour induction, compared with ERCS, removing covariate maternal BMI from the analysis.
**Table S16**. Outcomes according to actual mode of birth: planned and actual VBAC and planned VBAC but in‐labour non‐elective repeat caesarean section, compared with ERCS, removing covariate maternal BMI from the analysis.

Supplementary Material

Supplementary Material

Supplementary Material
