# Supplementary material for: Planned mode of birth after previous caesarean section and special educational needs in childhood: a population‐based record linkage cohort study
Source: BJOG. 2021 Jul 28;128(13):2158–68. doi: 10.1111/1471-0528.16828 (PMC9291107; doi:10.1111/1471-0528.16828)
Supplement: Supplementary file 1 — Figure S1. Conception framework of how sociodemographic, maternal medical and pregnancy‐related and infant‐related factors might influence the relationship between planned mode of birth after previous caesarean and special educational needs (SENs) in the child. Figure S2. Outcomes following: (A) planned VBAC, compared with ERCS; (B) planned VBAC without labour induction, compared with ERCS; (C) planned VBAC with labour induction, compared with ERCS; (D) planned and actual VBAC, compared with ERCS; and (E) planned VBAC but in‐labour non‐elective repeat caesarean section, compared with ERCS. Table S1. Data sources. Table S2. Data sources, codes and database fields used to identify study population, exposures, outcomes and covariates. Table S3. Characteristics of children included in the study compared with those not included because they did not link to any pupil census records between 2007 and 2016 when the child was aged 4–11 years and attending a primary or special school. Table S4. Outcomes according to actual mode of birth: planned and actually had VBAC and planned VBAC but had in‐labour non‐elective repeat caesarean section, compared with ERCS. Table S5. Complete case analysis of outcomes following planned VBAC, compared with ERCS. Table S6. Complete case analysis of outcomes following planned VBAC with and without labour induction, compared with ERCS. Table S7. Complete case analysis of outcomes according to actual mode of birth: planned and actual VBAC and planned VBAC but in‐labour non‐elective repeat caesarean section, compared with ERCS. Table S8. Outcomes following planned VBAC compared with ERCS at ≥ 39 weeks of gestation. Table S9. Outcomes following planned VBAC with and without labour induction, compared with ERCS, at ≥39 weeks of gestation. Table S10. Outcomes according to actual mode of birth: planned and actual VBAC and planned VBAC but in‐labour non‐elective repeat caesarean section, compared with ERCS, at ≥39 weeks of gestation. Table S11. Outcome [file BJO-128-2158-s003.docx]

**Supplementary online content**

**Table S1. Data sources**

| **Data source^a^** | **Description of data source** | **Completeness and quality of data source** |
| --- | --- | --- |
| National Records of  Scotland (NRS) live births and stillbirths | Statutory data on all live births and stillbirths in Scotland | Compiled from birth registrations, with registration mandated by law and subject to various quality checks^1^, considered to give most accurate measure of number of live and stillbirths in Scotland with data quality regarded as high^2^ |
| The Scottish Morbidity Record Maternity Inpatient and Day Case dataset (SMR02) | Demographic and clinical data on all hospital inpatient and day case discharges from obstetric specialties in the National Health Service (NHS) Scotland | Has had a national coverage of around 98% of all births in NRS since the late 1970s^2^ and data are subject to regular quality checks^3,4^ |
| The Scottish Morbidity Record General/Acute Inpatient and Day case dataset (SMR01) | Demographic and clinical data on all hospital inpatient and day case discharges from acute specialties in NHS Scotland | Thought to be 99% complete and subject to regular quality checks^5^ |
| NRS deaths | Statutory data on all deaths in Scotland | Compiled from death registrations, with registration mandated by law and subject to various quality checks^1^, considered to be complete and of high quality |
| The Child Health Surveillance Programme Pre-School system (CHSP-PS) | Records information, including infant feeding data, collected during child health reviews of pre-school children in Scotland. | Number of Scottish Health Boards using CHSP-PS increased over time, with infant feeding data reported to be of high quality^6^. |
| Pupil census | Annual census conducted near the start (September) of every school year of all children attending publicly funded schools in Scotland including local authority maintained or grant-aided primary, secondary and special schools. Special schools provide education to children with complex or specific needs which cannot be met in mainstream schools. Data was available from 2007-2016 (academic years 2007/2008-2016/2017) at the time it was obtained | Contains data on all children attending publicly funded schools in Scotland (~96% of all pupils in Scotland)^7^. Data subject to various quality assurance checks and is generally considered to be of high quality^8^ |

^a^The health data was linked using exact matching of the Community Health Index number and the health and education (pupil census) data was linked using probability matching techniques, previously shown to be 99% accurate for singleton children^9^.

**Table S2. Data sources, codes and database fields used to identify study population, exposures, outcomes and covariates**

|  | **Data source** | **Database fields/codes** |
| --- | --- | --- |
| **Inclusion criteria** |  |  |
| ≥ 1 previous caesarean sections | SMR02 | Previous caesarean sections field ≥1 OR at least one previous birth with a code for caesarean section in mode of delivery field (7 or 8) and/or an OPCS-4 or OPCS-3 code for caesarean section (R17-R18, R251, 764-766, 769), using SMR02 records going back to 1981 |
| Baby live born | NRS live births, stillbirths and deaths | Data source=NRS Births, checked against period of death field |
| Singleton birth | NRS live births and stillbirths | Numbirths field=1 |
| Term birth | SMR02 | Gestation at birth 37-41 completed weeks according to Estimated gestation field (containing number of completed weeks of gestation as judged by the clinician, usually on the basis of ultrasound) OR according to gestation derived from date of delivery and date of last menstrual period fields if Estimated gestation missing (0.04% of eligible births) |
| **Exclusion criteria** |  |  |
| Non-cephalic presentation | SMR02 | Delivery episodes with a code for breech or shoulder in presentation at delivery field (4 or 6) OR a code for breech delivery or breech extraction in mode of delivery field (5 or 6) OR an ICD-10 code for breech delivery or breech extraction (O801, O830-O831) OR an OPCS-4 code for breech delivery or breech extraction (R19-R20) OR an ICD-10 code for maternal care for malpresentation of fetus (0320-0322, 0326-0329) |
| Placenta praevia | SMR02 | Delivery episodes with an ICD-10 code for placenta praevia (O440-O441) |
| Abdominal pregnancy | SMR02 | Delivery episodes with an ICD-10 code for delivery of or maternal care for viable fetus in abdominal pregnancy (O833, O367) |
| Known or suspected disproportion of maternal and/or fetal origin | SMR02 | Delivery episodes with an ICD-10 code for maternal care for known or suspected disproportion (O33) |
| Tumour of corpus uteri | SMR02 | Delivery episodes with an ICD-10 code for maternal care for tumour of corpus uteri (O341) |
| Pre-labour non-elective caesarean section | SMR02 | Code for non-elective caesarean section in mode of delivery field (8) AND duration of labour field=0 |
| Child died before the age of 4 years old | NRS deaths | Age at death <4 years |
| **Exposures** |  |  |
| Elective repeat caesarean section (ERCS) | SMR02 | Code for elective caesarean section in mode of delivery field (7) in women with ≥ 1 previous caesarean sections |
| Planned vaginal birth after previous caesarean section (planned VBAC) | SMR02 | Code for vaginal birth in mode of delivery field (0, 1, 2, 3, 4, A, B, C, D or E) OR code for non-elective caesarean section in mode of delivery field (8) AND duration of labour field ≥1 hour in women with ≥1 previous caesarean sections |
| Planned VBAC without labour induction | SMR02 | Criteria for Planned VBAC AND code for none in induction of labour field (0) |
| Planned VBAC with labour induction | SMR02 | Criteria for Planned VBAC AND code for induction of labour using artificial rupture of membranes (ARM), oxytocics, ARM & oxytocics, prostaglandins, prostaglandins & ARM, prostaglandins & oxytocics, prostaglandins & ARM & oxytocics or other method in induction of labour field (1-8). Surgical induction defined as using ARM to induce labour and medical induction defined as using oxytocics and/or prostaglandins to induce labour. |
| Vaginal birth after previous caesarean section (VBAC) | SMR02 | Code for vaginal birth in mode of delivery field (0, 1, 2, 3, 4, A, B, C, D or E) in women with ≥ 1 previous caesarean sections |
| In-labour non-elective repeat caesarean section | SMR02 | Code for non-elective caesarean section in mode of delivery field (8) AND duration of labour field ≥1 hour in women with ≥1 previous caesarean sections |
| **Outcomes** |  |  |
| Any record of special educational needs (SENs) | Pupil census | Main analysis – any of the following codes in student need category/reason for support fields in any Pupil census year when child was aged 4-11 years and attending a primary or special school (student stage field P1-P7 or SP): 10 (learning disability), 11 (dyslexia), 12 (other specific learning difficulty), 13 (other moderate learning difficulty), 20 (visual impairment), 21 (hearing impairment), 22 (deafblind), 23 (physical or motor impairment), 24 (language or speech disorder), 25 (autistic spectrum disorder), 26 (social, emotional and behavioural difficulty), 27 (physical health problem), 28 (mental health problem). Detailed descriptions of each student need category/reason for support can be found in the School/Pupil Census Data Specification^10^  Sensitivity analysis – any of the above codes in student need category/reason for support fields at a particular Pupil census year when child was aged 4-11 years and attending a primary or special school (student stage field P1-P7 or SP), analysed as a repeated measures yearly outcome. |
| Learning disability | Pupil census | Main analysis – code for learning disability in student need category/reason for support fields (10) in any Pupil census year when child was aged 4-11 years and attending a primary or special school (student stage field P1-P7 or SP).  Sensitivity analysis – code for learning disability in student need category/reason for support fields (10) at a particular Pupil census year when child was aged 4-11 years and attending a primary or special school (student stage field P1-P7 or SP), analysed as a repeated measures yearly outcome. |
| Dyslexia | Pupil census | Main analysis – code for dyslexia in student need category/reason for support fields (11) in any Pupil census year when child was aged 4-11 years and attending a primary or special school (student stage field P1-P7 or SP).  Sensitivity analysis – code for dyslexia in student need category/reason for support fields (11) at a particular Pupil census year when child was aged 4-11 years and attending a primary or special school (student stage field P1-P7 or SP), analysed as a repeated measures yearly outcome. |
| Other learning difficulty | Pupil census | Main analysis – code for other specific or other moderate learning difficulty in student need category/reason for support fields (12 or 13) in any Pupil census year when child was aged 4-11 years and attending a primary or special school (student stage field P1-P7 or SP).  Sensitivity analysis – code for other specific or other moderate learning difficulty in student need category/reason for support fields (12 or 13) at a particular Pupil census year when child was aged 4-11 years and attending a primary or special school (student stage field P1-P7 or SP), analysed as a repeated measures yearly outcome. |
| Sensory impairment | Pupil census | Main analysis – code for visual impairment, hearing impairment or deafblind in student need category/reason for support fields (20-22) in any Pupil census year when child was aged 4-11 years and attending a primary or special school (student stage field P1-P7 or SP).  Sensitivity analysis – code for visual impairment, hearing impairment or deafblind in student need category/reason for support fields (20-22) at a particular Pupil census year when child was aged 4-11 years and attending a primary or special school (student stage field P1-P7 or SP), analysed as a repeated measures yearly outcome. |
| Physical or motor impairment | Pupil census | Main analysis – code for physical or motor impairment in student need category/reason for support fields (23) in any Pupil census year when child was aged 4-11 years and attending a primary or special school (student stage field P1-P7 or SP).  Sensitivity analysis – code for physical or motor impairment in student need category/reason for support fields (23) at a particular Pupil census year when child was aged 4-11 years and attending a primary or special school (student stage field P1-P7 or SP), analysed as a repeated measures yearly outcome. |
| Language or speech disorder | Pupil census | Main analysis – code for language or speech disorder in student need category/reason for support fields (24) in any Pupil census year when child was aged 4-11 years and attending a primary or special school (student stage field P1-P7 or SP).  Sensitivity analysis – code for language or speech disorder in student need category/reason for support fields (24) at a particular Pupil census year when child was aged 4-11 years and attending a primary or special school (student stage field P1-P7 or SP), analysed as a repeated measures yearly outcome. |
| Autistic spectrum disorder | Pupil census | Main analysis – code for autistic spectrum disorder in student need category/reason for support fields (25) in any Pupil census year when child was aged 4-11 years and attending a primary or special school (student stage field P1-P7 or SP).  Sensitivity analysis – code for autistic spectrum disorder in student need category/reason for support fields (25) at a particular Pupil census year when child was aged 4-11 years and attending a primary or special school (student stage field P1-P7 or SP), analysed as a repeated measures yearly outcome. |
| Social, emotional and behavioural difficulty or mental health problem | Pupil census | Main analysis – code for social, emotional and behavioural difficulty or mental health problem in student need category/reason for support fields (26 or 28) in any Pupil census year when child was aged 4-11 years and attending a primary or special school (student stage field P1-P7 or SP).  Sensitivity analysis – code for social, emotional and behavioural difficulty or mental health problem in student need category/reason for support fields (26 or 28) at a particular Pupil census year when child was aged 4-11 years and attending a primary or special school (student stage field P1-P7 or SP), analysed as a repeated measures yearly outcome. |
| Physical health problem | Pupil census | Main analysis – code for physical health problem in student need category/reason for support fields (27) in any Pupil census year when child was aged 4-11 years and attending a primary or special school (student stage field P1-P7 or SP).  Sensitivity analysis – code for physical health problem in student need category/reason for support fields (27) at a particular Pupil census year when child was aged 4-11 years and attending a primary or special school (student stage field P1-P7 or SP), analysed as a repeated measures yearly outcome. |
| **Socio-demographic maternal medical and pregnancy-related characteristics** |  |  |
| Maternal age | SMR02 | Derived from mother’s date of birth and date of delivery in current pregnancy |
| Mother’s country of birth | NRS live and stillbirths | Mother’s Country of Birth field |
| Marital status/registration type | NRS live and stillbirths | Parents married indicator field |
| Socioeconomic status | NRS live and stillbirths | Socioeconomic status of mother if sole registered birth or highest of mother or father’s socioeconomic status for births registered inside marriage or jointly registered by both parents outside marriage. Socioeconomic status defined by National Statistics Socio-Economic Classification (NS-SEC) based on occupation and employment status |
| Child’s ethnicity | Pupil Census | Ethnic background field. If child in more than one pupil census and had different known ethnic group value recorded in different census years, set to the child’s most commonly recorded ethnic group value |
| Number of pervious caesarean sections | SMR02 | Previous caesarean sections field. Number of previous caesarean sections according to previous caesarean sections field was cross-checked against woman’s previous delivery records in SMR02. Where this was found to be less than the number of previous caesarean sections observed to date (14% of eligible births), it was overwritten with the higher number. Also, if the previous caesarean sections field was missing (0.3% of eligible births), number of previous caesarean sections was derived from the number of previous caesarean sections observed to date. |
| Any prior vaginal birth | SMR02 | Derived from parity (number of previous pregnancies resulting in either a live birth or stillbirth) and number of previous caesarean sections. Woman’s previous delivery records were also examined for evidence of any prior vaginal births. |
| Inter-pregnancy interval | SMR02 | Derived from interval between date of delivery of current pregnancy and date of delivery of previous delivery minus gestational age at birth of current pregnancy |
| Maternal smoking status at booking | SMR02 | Booking smoking history field |
| Maternal body mass index (BMI) at booking | SMR02 | Derived from height and weight of mother at booking fields. Maternal height values < 120 cm and > 200 cm, weight of mother at booking values < 32kg and > 180 kg and maternal BMI at booking values ≤15 and >80 kg/m2 were considered to be implausible based on published values^11,12^. If height was missing or implausible, it was set to the median value observed in the woman’s other records in SMR02. Implausible values of maternal weight or BMI at booking were set to missing. |
| Any hypertensive disorder | SMR02 | Antenatal or delivery episodes with an ICD-10 code for pre-existing or gestational hypertensive disorder (O10-O11, O13-O16, I10) |
| Pre-existing or gestational diabetes mellitus | SMR02 | Antenatal or delivery episodes with a code for pre-existing or gestational diabetes mellitus in diabetes field (1-3) OR an ICD-10 code for pre-existing or gestational diabetes mellitus (O24, E10-E11) |
| Prelabour rupture of membranes | SMR02 | Antenatal or delivery episodes with an ICD-10 code for premature rupture of membranes (O42) |
| **Infant-related characteristics** |  |  |
| Gender of child | NRS live births and stillbirths | Sex (gender) field |
| Gestational age at birth | SMR02 | Estimated gestation field (containing number of completed weeks of gestation as judged by the clinician, usually on the basis of ultrasound) or if missing (0.04% of eligible births) derived from date of delivery and date of last menstrual period fields |
| Birth weight centile | SMR02 | Derived from gestational age at birth, birthweight and gender of child using sex-specific birth weight for gestational age centiles as reported by Bonellie et at^13^. Implausible birth weights for gestational age were identified as those more than twice the inter-quartile range below and above the first and third quartile, respectively, using the sex-specific birth weight for gestational age centiles reported by Bonellie et at^13^. Implausible values were set to missing. |
| **Other characteristics** |  |  |
| Adverse perinatal outcome (including admission to a neonatal unit, resuscitation requiring drugs and/or intubation or an Apgar score < 7 at 5 minutes) | SMR02 | Code for admitted in neonatal indicator field (1-2), code for bag and mask with drugs, intubation for IPVV with/without drugs or drugs only in resuscitation field (3-6), or < 7 in Apgar score field |
| Maternal intrapartum or postpartum complication (including uterine rupture, peripartum hysterectomy, blood transfusion, puerperal sepsis, other puerperal infection, surgical injury (damage to bowel, bladder or ureter requiring surgical repair) or third- or fourth-degree perineal tear) | SMR02 and SMR01 | Delivery episodes with an ICD-10 code for uterine rupture (O710-O711); OPCS-4 code for hysterectomy (Q071-Q075, Q08, R251) within 6 weeks of birth; Delivery episodes with an OPCS-4 code for blood transfusion (X331-X333, X337-X339, X341); ICD-10 code for puerperal sepsis (O85) within 6 weeks of delivery; ICD-10 code for other puerperal infections (O86) within 6 weeks of birth; OPCS-4 code for any of the following within 6 weeks of birth: total excision of colon and rectum, total excision of colon, extended excision of right hemicolon, other excision of right hemicolon, excision of transverse colon, excision of left hemicolon, excision of sigmoid colon, other excision of colon, exteriorisation of caecum, other exteriorisation of colon, subtotal excision of colon, exteriorisation of colon, repair of anus, other operations on the anal sphincter to control continence, excision of ureter, urinary diversion, replantation of ureter, other connection of ureter, repair of ureter, incision of ureter, other open operations on ureter,therapeutic nephroscopic operations on ureter, therapeutic ureteroscopic operations on ureter, other therapeutic endoscopic operations on ureter, percutaneous ureteric stent procedures, total excision of bladder, partial excision of bladder, enlargement of bladder, other repair of bladder or open drainage of bladder (H04-H11, H14-H15, H29, H32, H50, H57, M18-M23, M25-M27, M29, M33-M38); Delivery episodes with a code for third- or fourth-degree tear in tears field (3 or 4) OR ICD-10 code for third- or fourth-degree perineal laceration during delivery (O702-0703) OR OPCS-4 code for repair of obstetric laceration of perineum and sphincter of anus or repair of obstetric laceration of perineum and sphincter and mucosa of anus (R322, R325) |
| Any breastfeeding at 6-8 week review | CHSP-PS | Method of feeding at 6-8 week review breast milk only or mixed breast and formula milk |

**Socio-demographic characteristics**

maternal age, country of birth, ethnicity, marital status, socio-economic status

**Maternal medical and pregnancy-related characteristics**

number of previous caesarean sections, any prior vaginal birth, interpregnancy interval, maternal smoking, maternal body mass index (BMI), maternal pre-existing or gestational complications

**Planned mode of birth after previous caesarean section**

**Special educational needs in the child**

**Factors potentially on causal pathway**

**Infant-related characteristics**

Infant gender, gestational age, birth weight centile

**Adverse perinatal outcome** e.g. admission to a neonatal unit

**Breastfeeding**

**Maternal intrapartum or postpartum complications/morbidity** e.g. uterine rupture

**Figure S1. Conception framework of how socio-demographic, maternal medical and pregnancy-related and infant-related factors might influence the relationship between planned mode of birth after previous caesarean and special educational needs (SENs) in the child**

Note, arrows are shown at both ends of line between planned mode of birth and infant-related factors as gestational age is considered as a potential confounder and a potential mediator in that gestational age may influence planned mode of birth (e.g. if a woman has not gone into spontaneous labour by 40-41 weeks’ gestation she may choose an ERCS rather than risk a planned VBAC with induction of labour) but planned mode of birth may also influence gestational age (since 2004, UK guidelines^14,15^ have recommended that an ERCS should be performed from 39 weeks’ gestation, which typically results in women having an ERCS giving birth at an earlier gestational age compared to women awaiting spontaneous onset of labour). Earlier gestational age at birth, even at term, in turn is a risk factor for adverse perinatal^16-18^ and child outcomes^19,20^ and may also influence the likelihood of breastfeeding^21^

**Table S3. Characteristics of children included in the study compared to those not included because they did not link to any pupil census records between 2007-2016 when child was aged 4-11 years and attending a primary or special school**

|  | **Not included in study as no pupil census records**  **No. (%)^a^ unless otherwise stated (n=5133)** | **Included in study No. (%)^a^ unless otherwise stated (n=44892)** | **P-value** |
| --- | --- | --- | --- |
| **Socio-demographic medical & pregnancy-related characteristics** |  |  |  |
| Maternal age (years) |  |  |  |
| Less than 25 | 411 (8.0) | 5207 (11.6) |  |
| 25-29 | 1078 (21.0) | 10009 (22.3) |  |
| 30-34 | 1881 (36.6) | 15535 (34.6) |  |
| 35-39 | 1407 (27.4) | 11628 (25.9) |  |
| 40 or more | 356 (6.9) | 2513 (5.6) | <0.001 |
| Median (IQR) maternal age (years) | 33 (29-36) | 32 (28-35) | <0.001 |
| Mother’s country of birth |  |  |  |
| UK | 3719 (72.5) | 41194 (91.8) |  |
| Non-UK | 1414 (27.5) | 3698 (8.2) | <0.001 |
| Marital status/registration type |  |  |  |
| Married or joint registration same address | 4757 (92.7) | 40174 (89.5) |  |
| Joint registration different address | 221 (4.3) | 3103 (6.9) |  |
| Sole registration | 155 (3.0) | 1615 (3.6) | <0.001 |
| Socioeconomic status^b^ |  |  |  |
| Managerial & professional | 3140 (61.2) | 20572 (45.8) |  |
| Intermediate | 806 (15.7) | 10230 (22.8) |  |
| Routine & manual | 870 (16.9) | 12268 (27.3) |  |
| Other^c^ | 317 (6.2) | 1822 (4.1) | <0.001 |
| Planned mode of birth |  |  |  |
| Planned VBAC | 2130 (41.5) | 18851 (42.0) |  |
| ERCS | 3003 (58.5) | 26041 (58.0) | 0.495 |
| Number of prior caesarean sections |  |  |  |
| 1 | 4258 (83.0) | 37545 (83.6) |  |
| 2 or more | 875 (17.0) | 7347 (16.4) | 0.213 |
| Median (IQR) number of previous caesarean sections | 1 (1-1) | 1 (1-1) | 0.178 |
| Any prior vaginal birth^d^ |  |  |  |
| No | 3806 (74.7) | 33163 (74.2) |  |
| Yes | 1291 (25.3) | 11525 (25.8) | 0.475 |
| Parity^d^ |  |  |  |
| 1 | 3082 (60.6) | 27463 (61.5) |  |
| 2 or more | 2008 (39.4) | 17164 (38.5) | 0.170 |
| Median (IQR) parity | 1 (1-2) | 1 (1-2) | 0.125 |
| Inter-pregnancy interval (months)^d^ |  |  |  |
| 24 or more | 1880 (49.0) | 24264 (58.9) |  |
| 12-23 | 1262 (32.9) | 10768 (26.1) |  |
| Less than 12 | 693 (18.1) | 6152 (14.9) | <0.001 |
| Median (IQR) inter-pregnancy interval (months)^d^ | 23.7 (14.4-39.9) | 28.7 (16.5-49.5) | <0.001 |
| Mother smoked at booking^c^ |  |  |  |
| No | 3989 (86.1) | 32282 (79.3) |  |
| Yes | 643 (13.9) | 8427 (20.7) | <0.001 |
| Maternal BMI at booking (Kg/m2)^d^ |  |  |  |
| Less than 25 | 1254 (44.0) | 10389 (39.3) |  |
| 25-29.9 | 868 (30.5) | 8219 (31.1) |  |
| 30 or more | 725 (25.5) | 7808 (29.6) | <0.001 |
| Median (IQR) BMI at booking (Kg/m2)^d^ | 25.8 (22.8-30.1) | 26.4 (23.3-31.2) | <0.001 |
| Hypertensive disorder | 270 (5.3) | 2819 (6.3) | 0.004 |
| Diabetes | 113 (2.2) | 1096 (2.4) | 0.289 |
| Prelabour rupture of membranes | 187 (3.6) | 1751 (3.9) | 0.365 |
| **Infant-related characteristics** |  |  |  |
| Male infant | 2649 (51.6) | 22889 (51.0) | 0.400 |
| Gestational age at birth (weeks) |  |  |  |
| 39-41 | 3677 (71.6) | 32289 (71.9) |  |
| 37-38 | 1456 (28.4) | 12603 (28.1) | 0.660 |
| Median (IQR) gestational age at birth (weeks) | 39 (38-40) | 39 (38-40) | 0.441 |
| Birth weight centile^d^ |  |  |  |
| 10-90th | 4105 (80.4) | 35415 (79.3) |  |
| Less than 10th | 397 (7.8) | 3547 (7.9) |  |
| More than 90th | 605 (11.8) | 5709 (12.8) | 0.135 |
| **Other characteristics** |  |  |  |
| Adverse perinatal outcome^d,e^ |  |  |  |
| No | 4178 (92.6) | 36045 (92.7) |  |
| Yes | 324 (7.4) | 2854 (7.3) | 0.873 |
| Maternal intrapartum or postpartum complication^f^ |  |  |  |
| No | 4899 (95.4) | 42856 (95.5) |  |
| Yes | 234 (4.6) | 2036 (4.5) | 0.939 |
| Any breastfeeding at 6-8 wk review^d^ |  |  |  |
| No | 1756 (44.5) | 23787 (64.0) |  |
| Yes | 2190 (55.5) | 13381 (36.0) | <0.001 |

aPercentage of those with complete data.
bSocioeconomic status of mother for sole registered birth or highest of mother’s or father’s socioeconomic status for births registered inside marriage or jointly registered by both parents outside marriage. Socioeconomic status defined by NS-SEC based on occupation and employment status.

cOther includes never worked/long-term unemployed, student, not stated, or not classifiable.

^d^Missing data: any prior vaginal birth 240 (0.48%); parity 308 (0.62%); interpregnancy interval 5,006 (10.01%); maternal smoking status 4,684 (9.36%); maternal BMI 20,762 (41.50%); birthweight centile 247 (0.49%); adverse perinatal outcome 6,614 (13.22); any breastfeeding at 6-8 weeks 8,911 (17.81%)

^e^Adverse perinatal outcome includes admission to a neonatal unit, resuscitation with drugs and/or intubation or an Apgar score < 7 at 5 minutes.

^f^Intrapartum or postpartum complication includes uterine rupture, peripartum hysterectomy, blood transfusion, puerperal sepsis, other puerperal infection, surgical injury (damage to bowel, bladder or ureter requiring surgical repair), or third- or fourth-degree perineal tear

Abbreviations: BMI, body mass index; ERCS, elective repeat caesarean section; IQR, interquartile range; NS-SEC, National Statistics Socio-Economic Classification; VBAC, vaginal birth after previous caesarean

**
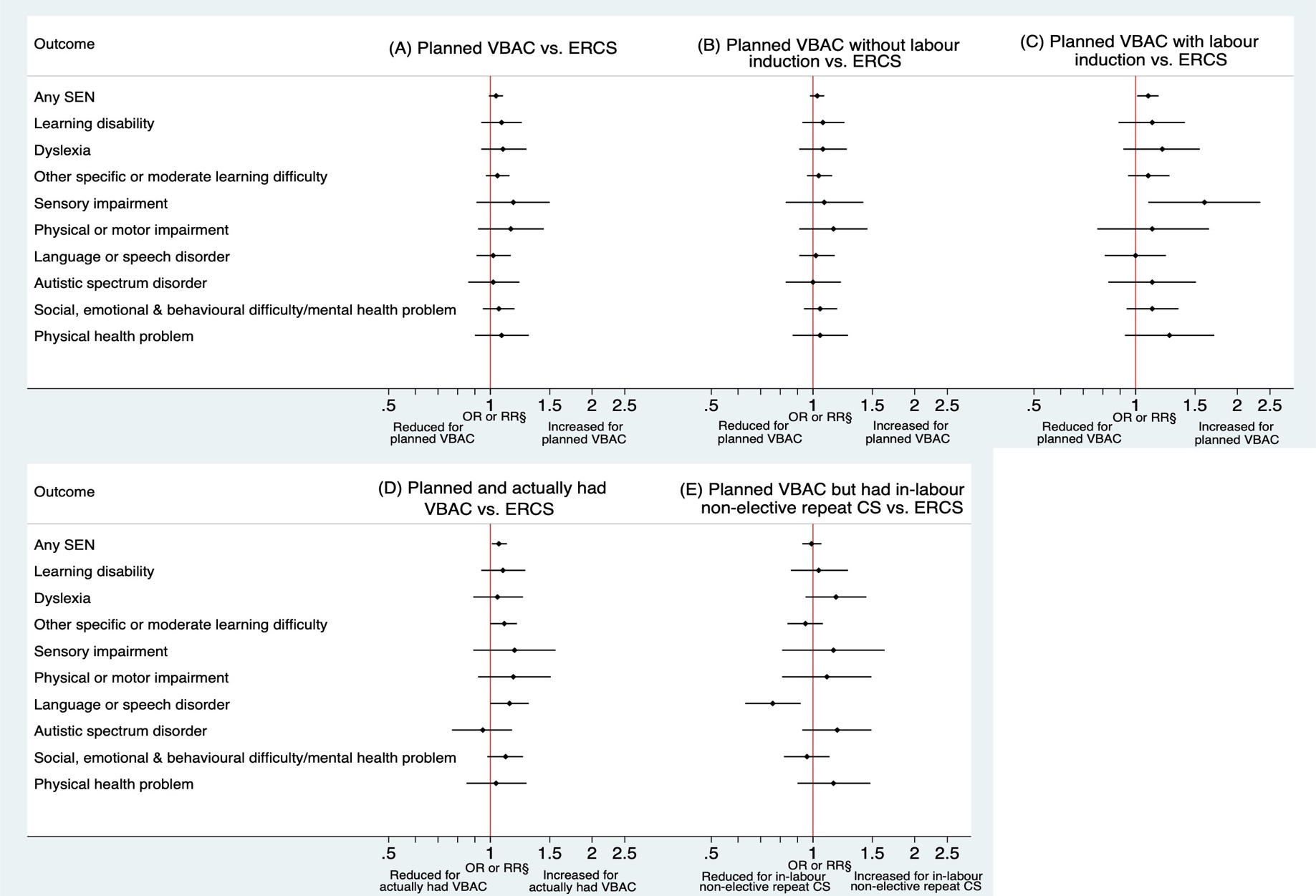
**

**Figure S2. Outcomes following (A) planned VBAC compared to ERCS, (B) planned VBAC without labour induction compared to ERCS, (C) planned VBAC with labour induction compared to ERCS, (D) planned and actually had VBAC compared to ERCS, (E) planned VBAC but had in-labour non-elective repeat caesarean section compared to ERCS**

§Effect estimates (ORs or RRs) adjusted for year of birth, socio-demographic (maternal age, mother's country of birth, marital status and socioeconomic status, child’s ethnicity), maternal medical and pregnancy-related factors (number of previous caesarean sections, any prior vaginal birth, inter-pregnancy interval, maternal smoking status at booking, maternal BMI at booking, hypertensive disorder, diabetes, and prelabour rupture of membranes), infant-related factors (sex of infant, gestational age at birth and birth weight centile) and breastfeeding at 6-8 weeks postpartum.

Abbreviations: BMI, body mass index; CS, caesarean section; ERCS, elective repeat caesarean section; OR, odds ratio; RR, risk ratio; SEN, special educational need; VBAC, vaginal birth after previous caesarean

**Table S4. Outcomes according to actual mode of birth – planned and actually had VBAC and planned VBAC but had in-labour non-elective repeat** **caesarean section compared to ERCS**

| **Outcomes** | **ERCS** | **Planned and actually had VBAC** | | | | |  | **Planned VBAC but had in-labour non-elective repeat caesarean section** | | | | |
| --- | --- | --- | --- | --- | --- | --- | --- | --- | --- | --- | --- | --- |
|  | **No. (%) of events (n=26041)** | **No. (%) of events (n=13564)** | **Base model^a^ OR or RR (95% CI)** | **Model A^b^ OR or RR (95% CI)** | **Model B^c^ OR or RR (95% CI)** | **Model C^d^ OR or RR (95% CI)** |  | **No. (%) of events (n=5287)** | **Base model^a^ OR or RR (95% CI)** | **Model A^b^ OR or RR (95% CI)** | **Model B^c^ OR or RR (95% CI)** | **Model C^d^ OR or RR (95% CI)** |
| Any SENs | 4592 | 2661 | **1.05** | 1.04 | **1.06** | **1.06** |  | 966 | 0.96 | 0.99 | 0.99 | 0.99 |
|  | (17.63) | (19.62) | **(1.01-1.10)** | (0.99-1.09) | **(1.01-1.11)** | **(1.01-1.12)** |  | (18.27) | (0.91-1.03) | (0.92-1.05) | (0.93-1.06) | (0.93-1.06) |
|  |  |  | **P=0.026** | P=0.140 | **P=0.031** | **P=0.021** |  |  | P=0.249 | P=0.623 | P=0.750 | P=0.820 |
| Learning disability | 712 | 426 | 1.05 | 1.04 | 1.08 | 1.09 |  | 155 | 0.94 | 0.99 | 1.03 | 1.04 |
|  | (2.73) | (3.14) | (0.93-1.19) | (0.90-1.20) | (0.93-1.25) | (0.94-1.27) |  | (2.93) | (0.79-1.13) | (0.82-1.19) | (0.85-1.25) | (0.86-1.27) |
|  |  |  | P=0.462 | P=0.634 | P=0.332 | P=0.242 |  |  | P=0.535 | P=0.906 | P=0.766 | P=0.683 |
| Dyslexia | 491 | 298 | 0.98 | 1.06 | 1.07 | 1.05 |  | 142 | 1.12 | 1.20 | 1.19 | 1.17 |
|  | (1.89) | (2.20) | (0.84-1.14) | (0.90-1.25) | (0.90-1.27) | (0.89-1.25) |  | (2.69) | (0.93-1.36) | (0.98-1.47) | (0.96-1.46) | (0.95-1.44) |
|  |  |  | P=0.769 | P=0.502 | P=0.435 | P=0.547 |  |  | P=0.234 | P=0.071 | P=0.107 | P=0.134 |
| Other specific or moderate learning difficulty | 2064 | 1229 | 1.07 | 1.06 | 1.09 | **1.10** |  | 415 | **0.89** | 0.93 | 0.95 | 0.95 |
|  | (7.93) | (9.06) | (0.99-1.15) | (0.97-1.16) | (1.00-1.19) | **(1.00-1.20)** |  | (7.85) | **(0.80-0.99)** | (0.83-1.04) | (0.84-1.07) | (0.84-1.07) |
|  |  |  | P=0.101 | P=0.184 | P=0.062 | **P=0.046** |  |  | **P=0.038** | P=0.219 | P=0.364 | P=0.411 |
| Sensory impairment | 204 | 121 | 1.08 | 1.12 | 1.19 | 1.18 |  | 44 | 0.99 | 1.07 | 1.16 | 1.15 |
|  | (0.78) | (0.89) | (0.85-1.36) | (0.85-1.46) | (0.90-1.58) | (0.89-1.56) |  | (0.83) | (0.70-1.39) | (0.76-1.52) | (0.81-1.65) | (0.81-1.63) |
|  |  |  | P=0.521 | P=0.428 | P=0.210 | P=0.242 |  |  | P=0.943 | P=0.689 | P=0.422 | P=0.448 |
| Physical or motor impairment | 259 | 147 | 1.04 | 1.10 | 1.17 | 1.17 |  | 57 | 1.02 | 1.04 | 1.09 | 1.10 |
|  | (0.99) | (1.08) | (0.85-1.27) | (0.86-1.40) | (0.91-1.50) | (0.92-1.51) |  | (1.08) | (0.76-1.36) | (0.78-1.40) | (0.80-1.48) | (0.81-1.49) |
|  |  |  | P=0.711 | P=0.452 | P=0.220 | P=0.206 |  |  | P=0.909 | P=0.786 | P=0.574 | P=0.560 |
| Language or speech disorder | 983 | 572 | **1.12** | **1.14** | 1.13 | **1.14** |  | 147 | **0.74** | **0.78** | **0.75** | **0.76** |
|  | (3.77) | (4.22) | **(1.01-1.25)** | **(1.01-1.29)** | (0.99-1.28) | **(1.00-1.30)** |  | (2.78) | **(0.62-0.88)** | **(0.65-0.94)** | **(0.63-0.91)** | **(0.63-0.92)** |
|  |  |  | **P=0.034** | **P=0.039** | P=0.069 | **P=0.047** |  |  | **P=0.001** | **P=0.007** | **P=0.003** | **P=0.004** |
| Autistic spectrum disorder | 419 | 198 | 0.89 | 0.91 | 0.95 | 0.95 |  | 104 | 1.19 | 1.18 | 1.18 | 1.18 |
|  | (1.61) | (1.46) | (0.75-1.06) | (0.75-1.11) | (0.78-1.16) | (0.77-1.16) |  | (1.97) | (0.96-1.49) | (0.94-1.48) | (0.93-1.49) | (0.93-1.49) |
|  |  |  | P=0.178 | P=0.349 | P=0.611 | P=0.590 |  |  | P=0.111 | P=0.163 | P=0.167 | P=0.173 |
| Social, emotional & behavioural difficulty or mental health problem | 1162 | 746 | **1.17** | 1.06 | 1.10 | 1.11 |  | 251 | 0.98 | 0.96 | 0.95 | 0.96 |
|  | (4.46) | (5.50) | **(1.06-1.29)** | (0.95-1.19) | (0.98-1.24) | (0.98-1.25) |  | (4.75) | (0.85-1.13) | (0.82-1.11) | (0.82-1.11) | (0.82-1.12) |
|  |  |  | **P=0.002** | P=0.307 | P=0.118 | P=0.089 |  |  | P=0.778 | P=0.556 | P=0.532 | P=0.591 |
| Physical health problem | 381 | 197 | 0.95 | 1.02 | 1.04 | 1.04 |  | 88 | 1.07 | 1.10 | 1.15 | 1.15 |
|  | (1.46) | (1.45) | (0.79-1.13) | (0.83-1.24) | (0.85-1.27) | (0.85-1.28) |  | (1.66) | (0.85-1.35) | (0.87-1.41) | (0.90-1.47) | (0.90-1.48) |
|  |  |  | P=0.545 | P=0.860 | P=0.726 | P=0.682 |  |  | P=0.567 | P=0.426 | P=0.276 | P=0.261 |

^a^Base model adjusted for year of birth only.

^b^Model A adjusted for year of birth, socio-demographic (maternal age, mother's country of birth, marital status, socioeconomic status and child’s ethnicity) and maternal medical and pregnancy-related factors (number of previous caesarean sections, any prior vaginal birth, inter-pregnancy interval, maternal smoking status at booking, maternal BMI at booking, hypertensive disorder, diabetes and prelabour rupture of membranes).

^c^Model B, adjusted for variables in Model A and additionally adjusted for infant-related factors (sex of infant, gestational age at birth and birth weight centile).

^d^Model C, adjusted for variables in Model B and additionally adjusted for any breastfeeding at 6-8 weeks postpartum.

Bold text indicates statistically significant findings at the 5% level.

Abbreviations: BMI, body mass index; CI, confidence interval; ERCS, elective repeat caesarean section; OR, odds ratio; RR, risk ratio; SENs, special educational needs; VBAC, vaginal birth after previous caesarean

**Table S5. Complete case analysis of outcomes following planned VBAC compared to ERCS**

| **Outcomes** | **Base model^a^ OR or RR 95% CI)** | **Model A^b^ OR or RR (95% CI)** | **Model B^c^ OR or RR (95% CI)** | **Model C^d^ OR or RR (95% CI)** |
| --- | --- | --- | --- | --- |
| Any SENs | 1.03 (0.99-1.07) | 1.05 (0.99-1.13) | 1.06 (0.99-1.13) | 1.05 (0.97-1.13) |
|  | P=0.194 | P=0.108 | P=0.112 | P=0.253 |
| Learning disability | 1.02 (0.91-1.14) | 1.09 (0.88-1.36) | 1.14 (0.90-1.42) | 1.16 (0.90-1.48) |
|  | P=0.754 | P=0.416 | P=0.273 | P=0.252 |
| Dyslexia | 1.02 (0.89-1.17) | 0.98 (0.76-1.25) | 1.01 (0.78-1.30) | 0.94 (0.70-1.25) |
|  | P=0.765 | P=0.845 | P=0.959 | P=0.652 |
| Other specific or moderate learning difficulty | 1.02 (0.95-1.09) | 1.09 (0.97-1.22) | 1.10 (0.97-1.24) | 1.11 (0.97-1.28) |
|  | P=0.658 | P=0.164 | P=0.131 | P=0.129 |
| Sensory impairment | 1.05 (0.85-1.30) | 0.93 (0.65-1.34) | 1.03 (0.71-1.51) | 1.07 (0.72-1.60) |
|  | P=0.633 | P=0.709 | P=0.865 | P=0.732 |
| Physical or motor impairment | 1.03 (0.86-1.24) | 1.00 (0.71-1.40) | 1.15 (0.81-1.62) | 1.04 (0.71-1.54) |
|  | P=0.731 | P=0.996 | P=0.435 | P=0.829 |
| Language or speech disorder | 1.02 (0.92-1.12) | 1.13 (0.97-1.31) | 1.10 (0.93-1.29) | 1.11 (0.92-1.32) |
|  | P=0.762 | P=0.122 | P=0.262 | P=0.271 |
| Autistic spectrum disorder | 0.97 (0.84-1.13) | 1.01 (0.78-1.31) | 0.97 (0.74-1.27) | 0.90 (0.68-1.20) |
|  | P=0.732 | P=0.945 | P=0.827 | P=0.483 |
| Social, emotional and & behavioural difficulty or mental health problem | **1.12 (1.02-1.22)** | 1.11 (0.96-1.29) | 1.10 (0.94-1.29) | 1.04 (0.87-1.24) |
|  | **P=0.017** | P=0.157 | P=0.218 | P=0.647 |
| Physical health problem | 0.98 (0.84-1.15) | 1.16 (0.90-1.49) | 1.14 (0.88-1.48) | 1.12 (0.83-1.50) |
|  | P=0.822 | P=0.241 | P=0.315 | P=0.464 |

^a^Base model adjusted for year of birth only.

^b^Model A adjusted for year of birth, socio-demographic (maternal age, mother's country of birth, marital status, socioeconomic status and child’s ethnicity) and maternal medical and pregnancy-related factors (number of previous caesarean sections, any prior vaginal birth, inter-pregnancy interval, maternal smoking status at booking, maternal BMI at booking, hypertensive disorder, diabetes and prelabour rupture of membranes).

^c^Model B, adjusted for variables in Model A and additionally adjusted for infant-related factors (sex of infant, gestational age at birth and birth weight centile).

^d^Model C, adjusted for variables in Model B and additionally adjusted for any breastfeeding at 6-8 weeks postpartum.

Bold text indicates statistically significant findings at the 5% level.

Abbreviations: BMI, body mass index; CI, confidence interval; ERCS, elective repeat caesarean section; OR, odds ratio; RR, risk ratio; SENs, special educational needs; VBAC, vaginal birth after previous caesarean

| **Outcomes** | **Planned VBAC without labour induction vs. ERCS** | | | |  | **Planned VBAC with labour induction vs. ERCS** | | | |
| --- | --- | --- | --- | --- | --- | --- | --- | --- | --- |
|  | **Base model^a^ OR or RR (95% CI)** | **Model A^b^ OR or RR (95% CI)** | **Model B^c^ OR or RR (95% CI)** | **Model C^d^ OR or RR (95% CI)** |  | **Base model^a^ OR or RR (95% CI)** | **Model A^b^ OR or RR (95% CI)** | **Model B^c^ OR or RR (95% CI)** | **Model C^d^ OR or RR (95% CI)** |
| Any SENs | 1.01 | 1.04 | 1.04 | 1.03 |  | **1.13** | 1.12 | **1.15** | 1.13 |
|  | (0.96-1.05) | (0.98-1.12) | (0.97-1.12) | (0.96-1.12) |  | **(1.05-1.21)** | (1.00-1.27) | **(1.01-1.30)** | (0.99-1.30) |
|  | P=0.767 | P=0.205 | P=0.225 | P=0.412 |  | **P=0.001** | P=0.056 | **P=0.028** | P=0.073 |
| Learning disability | 1.01 | 1.13 | 1.16 | 1.18 |  | 1.09 | 0.91 | 1.00 | 1.03 |
|  | (0.89-1.14) | (0.90-1.41) | (0.92-1.46) | (0.92-1.52) |  | (0.89-1.33) | (0.61-1.37) | (0.66-1.53) | (0.65-1.64) |
|  | P=0.910 | P=0.281 | P=0.213 | P=0.203 |  | P=0.432 | P=0.665 | P=0.984 | P=0.900 |
| Dyslexia | 1.00 | 0.99 | 1.02 | 0.93 |  | 1.11 | 0.84 | 0.88 | 0.91 |
|  | (0.86-1.15) | (0.76-1.29) | (0.78-1.33) | (0.69-1.25) |  | (0.88-1.40) | (0.51-1.39) | (0.53-1.47) | (0.52-1.58) |
|  | P=0.955 | P=0.952 | P=0.886 | P=0.641 |  | P=0.361 | P=0.502 | P=0.630 | P=0.737 |
| Other specific or moderate learning difficulty | 1.00 | 1.07 | 1.08 | 1.09 |  | 1.11 | 1.22 | **1.26** | **1.29** |
|  | (0.92-1.07) | (0.94-1.20) | (0.95-1.22) | (0.94-1.26) |  | (0.98-1.27) | (0.98-1.52) | **(1.01-1.57)** | **(1.00-1.65)** |
|  | P=0.928 | P=0.303 | P=0.244 | P=0.238 |  | P=0.097 | P=0.069 | **P=0.043** | **P=0.048** |
| Sensory impairment | 0.97 | 0.86 | 0.95 | 0.98 |  | 1.38 | 1.36 | 1.63 | 1.74 |
|  | (0.77-1.22) | (0.58-1.27) | (0.64-1.41) | (0.64-1.49) |  | (0.97-1.97) | (0.75-2.49) | (0.87-3.05) | (0.90-3.37) |
|  | P=0.794 | P=0.444 | P=0.791 | P=0.913 |  | P=0.072 | P=0.312 | P=0.127 | P=0.098 |
| Physical or motor impairment | 1.03 | 1.00 | 1.13 | 0.99 |  | 1.01 | 1.00 | 1.28 | 1.46 |
|  | (0.85-1.26) | (0.71-1.43) | (0.79-1.62) | (0.65-1.49) |  | (0.71-1.44) | (0.53-1.88) | (0.67-2.44) | (0.74-2.89) |
|  | P=0.734 | P=0.983 | P=0.501 | P=0.958 |  | P=0.947 | P=0.996 | P=0.451 | P=0.273 |
| Language or speech disorder | 1.01 | 1.13 | 1.09 | 1.09 |  | 1.06 | 1.13 | 1.11 | 1.22 |
|  | (0.90-1.12) | (0.96-1.32) | (0.93-1.29) | (0.90-1.31) |  | (0.88-1.28) | (0.85-1.51) | (0.81-1.51) | (0.88-1.71) |
|  | P=0.895 | P=0.142 | P=0.287 | P=0.376 |  | P=0.528 | P=0.404 | P=0.512 | P=0.237 |
| Autistic spectrum disorder | 0.95 | 1.05 | 1.00 | 0.91 |  | 1.09 | 0.81 | 0.79 | 0.86 |
|  | (0.81-1.11) | (0.80-1.36) | (0.76-1.31) | (0.68-1.23) |  | (0.83-1.45) | (0.48-1.39) | (0.46-1.37) | (0.49-1.52) |
|  | P=0.519 | P=0.736 | P=0.998 | P=0.534 |  | P=0.522 | P=0.448 | P=0.405 | P=0.608 |
| Social, emotional & behavioural difficulty or mental health problem | 1.09 | 1.11 | 1.09 | 1.05 |  | **1.26** | 1.17 | 1.20 | 1.03 |
|  | (0.99-1.20) | (0.95-1.29) | (0.93-1.28) | (0.87-1.26) |  | **(1.08-1.48)** | (0.89-1.54) | (0.90-1.60) | (0.74-1.43) |
|  | P=0.078 | P=0.192 | P=0.283 | P=0.612 |  | **P=0.004** | P=0.259 | P=0.207 | P=0.873 |
| Physical health problem | 0.95 | 1.10 | 1.09 | 1.06 |  | 1.16 | 1.53 | 1.55 | 1.53 |
|  | (0.80-1.12) | (0.85-1.44) | (0.83-1.43) | (0.78-1.44) |  | (0.88-1.53) | (1.00-2.35) | (0.99-2.42) | (0.93-2.53) |
|  | P=0.526 | P=0.460 | P=0.544 | P=0.707 |  | P=0.290 | P=0.052 | P=0.056 | P=0.098 |

**Table S6. Complete case analysis of outcomes following planned VBAC with and without labour induction compared to ERCS**

^a^Base model adjusted for year of birth only.

^b^Model A adjusted for year of birth, socio-demographic (maternal age, mother's country of birth, marital status, socioeconomic status and child’s ethnicity) and maternal medical and pregnancy-related factors (number of previous caesarean sections, any prior vaginal birth, inter-pregnancy interval, maternal smoking status at booking, maternal BMI at booking, hypertensive disorder, diabetes and prelabour rupture of membranes).

^c^Model B, adjusted for variables in Model A and additionally adjusted for infant-related factors (sex of infant, gestational age at birth and birth weight centile).

^d^Model C, adjusted for variables in Model B and additionally adjusted for any breastfeeding at 6-8 weeks postpartum.

Bold text indicates statistically significant findings at the 5% level.

Abbreviations: BMI, body mass index; CI, confidence interval; ERCS, elective repeat caesarean section; OR, odds ratio; RR, risk ratio; SENs, special educational needs; VBAC, vaginal birth after previous caesarean

**Table S7. Complete case analysis of outcomes according to actual mode of birth – planned and actually had a VBAC and planned VBAC but had in-labour non-elective repeat caesarean section compared to ERCS**

| **Outcomes** | **Planned and actually had VBAC vs. ERCS** | | | |  | **Planned VBAC but had in-labour non-elective repeat caesarean section vs. ERCS** | | | |
| --- | --- | --- | --- | --- | --- | --- | --- | --- | --- |
|  | **Base model^a^ OR or RR (95% CI)** | **Model A^b^ OR or RR (95% CI)** | **Model B^c^ OR or RR (95% CI)** | **Model C^d^ OR or RR (95% CI)** |  | **Base model^a^ OR or RR (95% CI)** | **Model A^b^ OR or RR (95% CI)** | **Model B^c^ OR or RR (95% CI)** | **Model C^d^ OR or RR (95% CI)** |
| Any SENs | **1.05** | 1.07 | **1.08** | 1.07 |  | 0.96 | 1.01 | 1.00 | 0.98 |
|  | **(1.01-1.10)** | (1.00-1.15) | **(1.00-1.16)** | (0.99-1.16) |  | (0.91-1.03) | (0.91-1.12) | (0.90-1.11) | (0.87-1.10) |
|  | **P=0.026** | P=0.063 | **P=0.043** | P=0.105 |  | P=0.249 | P=0.803 | P=0.956 | P=0.727 |
| Learning disability | 1.05 | 1.07 | 1.12 | 1.12 |  | 0.94 | 1.15 | 1.19 | 1.24 |
|  | (0.93-1.19) | (0.85-1.36) | (0.87-1.42) | (0.85-1.48) |  | (0.79-1.13) | (0.83-1.59) | (0.85-1.65) | (0.87-1.77) |
|  | P=0.462 | P=0.547 | P=0.380 | P=0.406 |  | P=0.535 | P=0.413 | P=0.316 | P=0.230 |
| Dyslexia | 0.98 | 0.96 | 1.00 | 0.99 |  | 1.12 | 1.02 | 1.03 | 0.81 |
|  | (0.84-1.14) | (0.72-1.27) | (0.75-1.32) | (0.72-1.36) |  | (0.93-1.36) | (0.71-1.48) | (0.70-1.50) | (0.52-1.28) |
|  | P=0.769 | P=0.755 | P=0.991 | P=0.955 |  | P=0.234 | P=0.909 | P=0.896 | P=0.371 |
| Other specific or moderate learning difficulty | 1.07 | 1.13 | **1.15** | 1.15 |  | **0.89** | 0.96 | 0.96 | 1.01 |
|  | (0.99-1.15) | (1.00-1.29) | **(1.01-1.32)** | (0.99-1.34) |  | **(0.80-0.99)** | (0.79-1.16) | (0.79-1.17) | (0.81-1.26) |
|  | P=0.101 | P=0.053 | **P=0.036** | P=0.061 |  | **P=0.038** | P=0.678 | P=0.695 | P=0.942 |
| Sensory impairment | 1.08 | 0.90 | 1.00 | 1.05 |  | 0.99 | 1.03 | 1.14 | 1.14 |
|  | (0.85-1.36) | (0.59-1.36) | (0.65-1.53) | (0.67-1.65) |  | (0.70-1.39) | (0.61-1.76) | (0.66-1.95) | (0.64-2.02) |
|  | P=0.521 | P=0.612 | P=0.984 | P=0.836 |  | P=0.943 | P=0.904 | P=0.643 | P=0.665 |
| Physical or motor impairment | 1.04 | 1.02 | 1.19 | 1.06 |  | 1.02 | 0.95 | 1.04 | 1.01 |
|  | (0.85-1.27) | (0.70-1.49) | (0.81-1.75) | (0.68-1.64) |  | (0.76-1.36) | (0.57-1.58) | (0.62-1.76) | (0.57-1.80) |
|  | P=0.711 | P=0.911 | P=0.372 | P=0.805 |  | P=0.909 | P=0.835 | P=0.878 | P=0.961 |
| Language or speech disorder | **1.12** | **1.20** | 1.18 | 1.21 |  | **0.74** | 0.92 | 0.88 | 0.85 |
|  | **(1.01-1.25)** | **(1.02-1.42)** | (0.99-1.40) | (0.99-1.46) |  | **(0.62-0.88)** | (0.70-1.20) | (0.67-1.16) | (0.63-1.15) |
|  | **P=0.034** | **P=0.029** | P=0.065 | P=0.058 |  | **P=0.001** | P=0.521 | P=0.360 | P=0.297 |
| Autistic spectrum disorder | 0.89 | 0.89 | 0.86 | 0.76 |  | 1.19 | 1.33 | 1.23 | 1.24 |
|  | (0.75-1.06) | (0.66-1.20) | (0.64-1.18) | (0.55-1.07) |  | (0.96-1.49) | (0.94-1.89) | (0.86-1.77) | (0.85-1.81) |
|  | P=0.178 | P=0.429 | P=0.353 | P=0.118 |  | P=0.111 | P=0.104 | P=0.251 | P=0.270 |
| Social, emotional & behavioural difficulty or mental health problem | **1.17** | 1.12 | 1.12 | 1.07 |  | 0.98 | 1.09 | 1.05 | 0.98 |
|  | **(1.06-1.29)** | (0.95-1.32) | (0.94-1.33) | (0.88-1.30) |  | (0.85-1.13) | (0.87-1.38) | (0.83-1.34) | (0.75-1.29) |
|  | **P=0.002** | P=0.172 | P=0.189 | P=0.525 |  | P=0.778 | P=0.449 | P=0.668 | P=0.893 |
| Physical health problem | 0.95 | 1.13 | 1.11 | 1.09 |  | 1.07 | 1.25 | 1.22 | 1.19 |
|  | (0.79-1.13) | (0.85-1.49) | (0.84-1.48) | (0.78-1.50) |  | (0.85-1.35) | (0.86-1.81) | (0.83-1.78) | (0.78-1.82) |
|  | P=0.545 | P=0.398 | P=0.464 | P=0.620 |  | P=0.567 | P=0.243 | P=0.310 | P=0.423 |

^a^Base model adjusted for year of birth only.

^b^Model A adjusted for year of birth, socio-demographic (maternal age, mother's country of birth, marital status, socioeconomic status and child’s ethnicity) and maternal medical and pregnancy-related factors (number of previous caesarean sections, any prior vaginal birth, inter-pregnancy interval, maternal smoking status at booking, maternal BMI at booking, hypertensive disorder, diabetes and prelabour rupture of membranes).

^c^Model B, adjusted for variables in Model A and additionally adjusted for infant-related factors (sex of infant, gestational age at birth and birth weight centile).

^d^Model C, adjusted for variables in Model B and additionally adjusted for any breastfeeding at 6-8 weeks postpartum.

Bold text indicates statistically significant findings at the 5% level.

Abbreviations: BMI, body mass index; CI, confidence interval; ERCS, elective repeat caesarean section; OR, odds ratio; RR, risk ratio; SENs, special educational needs; VBAC, vaginal birth after previous caesarean

**Table S8. Outcomes following planned VBAC compared to ERCS at ≥ 39 weeks’ gestation**

| **Outcomes** | **ERCS No. (%) of events (n=17110)** | **Planned VBAC No. (%) of events (n=15179)** | **Base model^a^ OR or RR (95% CI)** | **Model A^b^ OR or RR (95% CI)** | **Model B^c^ OR or RR (95% CI)** | **Model C^d^ OR or RR (95% CI)** |
| --- | --- | --- | --- | --- | --- | --- |
| Any SENs | 2788 (16.29) | 2818 (18.57) | 1.04 (0.99-1.10) | 1.02 (0.97-1.08) | 1.02 (0.97-1.08) | 1.03 (0.97-1.09) |
|  |  |  | P=0.088 | P=0.461 | P=0.437 | P=0.373 |
| Learning disability | 427 (2.50) | 439 (2.89) | 0.99 (0.86-1.14) | 0.97 (0.83-1.14) | 1.02 (0.86-1.21) | 1.04 (0.87-1.23) |
|  |  |  | P=0.888 | P=0.740 | P=0.803 | P=0.665 |
| Dyslexia | 291 (1.70) | 355 (2.34) | 1.04 (0.88-1.22) | 1.13 (0.94-1.35) | 1.14 (0.94-1.38) | 1.12 (0.93-1.36) |
|  |  |  | P=0.649 | P=0.196 | P=0.183 | P=0.236 |
| Other specific or moderate learning difficulty | 1237 (7.23) | 1281 (8.44) | 1.05 (0.96-1.14) | 1.01 (0.92-1.11) | 1.01 (0.91-1.12) | 1.02 (0.92-1.13) |
|  |  |  | P=0.301 | P=0.896 | P=0.848 | P=0.756 |
| Sensory impairment | 106 (0.62) | 123 (0.81) | 1.21 (0.92-1.58) | 1.15 (0.86-1.55) | 1.13 (0.82-1.55) | 1.11 (0.81-1.53) |
|  |  |  | P=0.171 | P=0.343 | P=0.472 | P=0.515 |
| Physical or motor impairment | 143 (0.84) | 149 (0.98) | 1.07 (0.85-1.35) | 1.10 (0.85-1.42) | 1.01 (0.77-1.34) | 1.01 (0.76-1.34) |
|  |  |  | P=0.552 | P=0.483 | P=0.920 | P=0.933 |
| Language or speech disorder | 619 (3.62) | 553 (3.64) | 1.02 (0.90-1.15) | 0.98 (0.86-1.11) | 0.93 (0.80-1.07) | 0.94 (0.81-1.08) |
|  |  |  | P=0.765 | P=0.728 | P=0.298 | P=0.394 |
| Autistic spectrum disorder | 249 (1.46) | 233 (1.54) | 1.03 (0.85-1.23) | 1.05 (0.85-1.29) | 1.04 (0.84-1.30) | 1.04 (0.83-1.29) |
|  |  |  | P=0.776 | P=0.648 | P=0.709 | P=0.735 |
| Social, emotional & behavioural difficulty or mental health problem | 711 (4.16) | 760 (5.01) | 1.10 (0.99-1.23) | 1.01 (0.90-1.14) | 1.03 (0.91-1.18) | 1.04 (0.92-1.19) |
|  |  |  | P=0.082 | P=0.864 | P=0.613 | P=0.519 |
| Physical health problem | 233 (1.36) | 222 (1.46) | 1.00 (0.83-1.20) | 1.09 (0.89-1.34) | 1.12 (0.89-1.40) | 1.13 (0.90-1.42) |
|  |  |  | P=0.973 | P=0.402 | P=0.322 | P=0.286 |

^a^Base model adjusted for year of birth only.

^b^Model A adjusted for year of birth, socio-demographic (maternal age, mother's country of birth, marital status, socioeconomic status and child’s ethnicity) and maternal medical and pregnancy-related factors (number of previous caesarean sections, any prior vaginal birth, inter-pregnancy interval, maternal smoking status at booking, maternal BMI at booking, hypertensive disorder, diabetes and prelabour rupture of membranes).

^c^Model B, adjusted for variables in Model A and additionally adjusted for infant-related factors (sex of infant, gestational age at birth and birth weight centile).

^d^Model C, adjusted for variables in Model B and additionally adjusted for any breastfeeding at 6-8 weeks postpartum.

Bold text indicates statistically significant findings at the 5% level.

Abbreviations: BMI, body mass index; CI, confidence interval; ERCS, elective repeat caesarean section; OR, odds ratio; RR, risk ratio; SENs, special educational needs; VBAC, vaginal birth after previous caesarean

**Table S9. Outcomes following planned VBAC with and without labour induction compared to ERCS at ≥ 39 weeks’ gestation**

| **Outcomes** | **ERCS** | **Planned VBAC without labour induction** | | | | |  | **Planned VBAC with labour induction** | | | | |
| --- | --- | --- | --- | --- | --- | --- | --- | --- | --- | --- | --- | --- |
|  | **No. (%) of events (n=17110)** | **No. (%) of events (n=12381)** | **Base model**^a^ **OR or RR (95% CI)** | **Model A^b^ OR or RR (95% CI)** | **Model B^c^ OR or RR (95% CI)** | **Model C^d^ OR or RR (95% CI)** |  | **No. (%) of events (n=2752)** | **Base model^a^ OR or RR (95% CI)** | **Model A^b^ OR or RR (95% CI)** | **Model B^c^ OR or RR (95% CI)** | **Model C^d^ OR or RR (95% CI)** |
| Any SENs | 2788 | 2238 | 1.02 | 1.01 | 1.01 | 1.02 |  | 572 | **1.14** | 1.05 | 1.06 | 1.06 |
|  | (16.29) | (18.08) | (0.97-1.08) | (0.96-1.07) | (0.96-1.07) | (0.96-1.08) |  | (20.78) | **(1.05-1.24)** | (0.96-1.14) | (0.97-1.16) | (0.97-1.16) |
|  |  |  | P=0.412 | P=0.730 | P=0.659 | P=0.575 |  |  | **P=0.001** | P=0.300 | P=0.191 | P=0.171 |
| Learning disability | 427 | 353 | 0.99 | 0.99 | 1.02 | 1.04 |  | 86 | 1.01 | 0.94 | 1.04 | 1.05 |
|  | (2.50) | (2.85) | (0.86-1.14) | (0.84-1.16) | (0.86-1.22) | (0.87-1.24) |  | (3.13) | (0.80-1.28) | (0.73-1.21) | (0.78-1.37) | (0.79-1.39) |
|  |  |  | P=0.882 | P=0.860 | P=0.799 | P=0.659 |  |  | P=0.937 | P=0.629 | P=0.807 | P=0.738 |
| Dyslexia | 291 | 276 | 1.01 | 1.10 | 1.12 | 1.10 |  | 77 | 1.13 | 1.21 | 1.23 | 1.22 |
|  | (1.70) | (2.23) | (0.85-1.20) | (0.91-1.33) | (0.92-1.36) | (0.90-1.34) |  | (2.80) | (0.87-1.46) | (0.92-1.60) | (0.92-1.67) | (0.90-1.65) |
|  |  | s | P=0.897 | P=0.327 | P=0.267 | P=0.337 |  |  | P=0.365 | P=0.170 | P=0.168 | P=0.194 |
| Other specific or moderate learning difficulty | 1237 | 1013 | 1.02 | 1.00 | 1.00 | 1.01 |  | 264 | **1.17** | 1.05 | 1.07 | 1.08 |
|  | (7.23) | (8.18) | (0.93-1.11) | (0.90-1.10) | (0.90-1.11) | (0.91-1.12) |  | (9.59) | **(1.01-1.35)** | (0.91-1.22) | (0.91-1.26) | (0.92-1.26) |
|  |  |  | P=0.689 | P=0.930 | P=0.984 | P=0.885 |  |  | **P=0.032** | P=0.496 | P=0.408 | P=0.375 |
| Sensory impairment | 106 | 92 | 1.11 | 1.07 | 1.05 | 1.04 |  | 30 | **1.58** | 1.52 | 1.50 | 1.49 |
|  | (0.62) | (0.74) | (0.84-1.48) | (0.78-1.47) | (0.76-1.47) | (0.75-1.46) |  | (1.09) | **(1.03-2.41)** | (0.97-2.37) | (0.92-2.45) | (0.91-2.43) |
|  |  |  | P=0.459 | P=0.666 | P=0.753 | P=0.805 |  |  | **P=0.035** | P=0.065 | P=0.106 | P=0.113 |
| Physical or motor impairment | 143 | 120 | 1.07 | 1.09 | 1.02 | 1.02 |  | 28 | 1.07 | 1.07 | 0.94 | 0.94 |
|  | (0.84) | (0.97) | (0.84-1.36) | (0.83-1.43) | (0.76-1.35) | (0.76-1.35) |  | (1.02) | (0.71-1.62) | (0.70-1.62) | (0.59-1.51) | (0.59-1.50) |
|  |  |  | P=0.606 | P=0.521 | P=0.903 | P=0.917 |  |  | P=0.740 | P=0.766 | P=0.806 | P=0.800 |
| Language or speech disorder | 619 | 450 | 1.01 | 0.98 | 0.94 | 0.95 |  | 101 | 1.05 | 0.95 | 0.88 | 0.89 |
|  | (3.62) | (3.63) | (0.89-1.15) | (0.86-1.13) | (0.81-1.08) | (0.82-1.10) |  | (3.67) | (0.84-1.30) | (0.76-1.18) | (0.69-1.12) | (0.69-1.13) |
|  |  |  | P=0.841 | P=0.822 | P=0.378 | P=0.491 |  |  | P=0.685 | P=0.623 | P=0.300 | P=0.341 |
| Autistic spectrum disorder | 249 | 181 | 0.98 | 1.02 | 1.02 | 1.01 |  | 52 | 1.26 | 1.21 | 1.22 | 1.21 |
|  | (1.46) | (1.46) | (0.81-1.19) | (0.82-1.27) | (0.81-1.28) | (0.81-1.27) |  | (1.89) | (0.92-1.71) | (0.87-1.68) | (0.86-1.72) | (0.86-1.72) |
|  |  |  | P=0.845 | P=0.869 | P=0.879 | P=0.908 |  |  | P=0.146 | P=0.259 | P=0.266 | P=0.275 |
| Social, emotional & behavioural difficulty or mental health problem | 711 | 597 | 1.07 | 1.00 | 1.02 | 1.03 |  | 163 | **1.27** | 1.07 | 1.13 | 1.14 |
|  | (4.16) | (4.82) | (0.95-1.20) | (0.88-1.14) | (0.89-1.17) | (0.90-1.18) |  | (5.92) | **(1.07-1.52)** | (0.89-1.29) | (0.91-1.39) | (0.92-1.40) |
|  |  |  | P=0.273 | P=0.990 | P=0.743 | P=0.641 |  |  | **P=0.008** | P=0.482 | P=0.263 | P=0.230 |
| Physical health problem | 233 | 173 | 0.96 | 1.06 | 1.09 | 1.10 |  | 49 | 1.19 | 1.25 | 1.34 | 1.35 |
|  | (1.36) | (1.40) | (0.78-1.17) | (0.85-1.32) | (0.86-1.37) | (0.87-1.39) |  | (1.78) | (0.87-1.63) | (0.90-1.73) | (0.94-1.93) | (0.94-1.95) |
|  |  |  | P=0.671 | P=0.591 | P=0.470 | P=0.423 |  |  | P=0.280 | P=0.184 | P=0.110 | P=0.102 |

^a^Base model adjusted for year of birth only.

^b^Model A adjusted for year of birth, socio-demographic (maternal age, mother's country of birth, marital status, socioeconomic status and child’s ethnicity) and maternal medical and pregnancy-related factors (number of previous caesarean sections, any prior vaginal birth, inter-pregnancy interval, maternal smoking status at booking, maternal BMI at booking, hypertensive disorder, diabetes and prelabour rupture of membranes).

^c^Model B, adjusted for variables in Model A and additionally adjusted for infant-related factors (sex of infant, gestational age at birth and birth weight centile).

^d^Model C, adjusted for variables in Model B and additionally adjusted for any breastfeeding at 6-8 weeks postpartum.

Bold text indicates statistically significant findings at the 5% level.

Abbreviations: BMI, body mass index; CI, confidence interval; ERCS, elective repeat caesarean section; OR, odds ratio; RR, risk ratio; SENs, special educational needs; VBAC, vaginal birth after previous caesarean

**Table S10. Outcomes according to actual mode of birth – planned and actually had a VBAC and planned VBAC but had in-labour non-elective repeat**

| **Outcomes** | **ERCS** | **Planned and actually had VBAC** | | | | |  | **Planned VBAC but had in-labour non-elective repeat caesarean section** | | | | |
| --- | --- | --- | --- | --- | --- | --- | --- | --- | --- | --- | --- | --- |
|  | **No. (%) of events (n=17110)** | **No. (%) of events (n=11049)** | **Base model^a^ OR or RR (95% CI)** | **Model A^b^ OR or RR (95% CI)** | **Model B^c^ OR or RR (95% CI)** | **Model C^d^ OR or RR (95% CI)** |  | **No. (%) of events (n=4130)** | **Base model^a^ OR or RR (95% CI)** | **Model A^b^ OR or RR (95% CI)** | **Model B^c^ OR or RR (95% CI)** | **Model C^d^ OR or RR (95% CI)** |
| Any SENs | 2788 | 2105 | **1.08** | 1.04 | 1.05 | 1.05 |  | 713 | 0.96 | 0.96 | 0.95 | 0.96 |
|  | (16.29) | (19.05) | **(1.02-1.13)** | (0.99-1.11) | (0.99-1.11) | (0.99-1.12) |  | (17.26) | (0.89-1.03) | (0.89-1.04) | (0.88-1.03) | (0.88-1.04) |
|  |  |  | **P=0.006** | P=0.145 | P=0.118 | P=0.093 |  |  | P=0.233 | P=0.323 | P=0.253 | P=0.284 |
| Learning disability | 427 | 330 | 1.04 | 1.00 | 1.05 | 1.06 |  | 109 | 0.87 | 0.91 | 0.97 | 0.98 |
|  | (2.50) | (2.99) | (0.89-1.20) | (0.85-1.19) | (0.87-1.25) | (0.89-1.28) |  | 2.64) | (0.70-1.08) | (0.72-1.14) | (0.76-1.23) | (0.77-1.25) |
|  |  |  | P=0.642 | P=0.980 | P=0.632 | P=0.508 |  |  | P=0.218 | P=0.423 | P=0.784 | P=0.871 |
| Dyslexia | 291 | 237 | 0.97 | 1.06 | 1.08 | 1.07 |  | 118 | 1.20 | **1.26** | 1.25 | 1.24 |
|  | (1.70) | (2.14) | (0.81-1.16) | (0.87-1.29) | (0.88-1.33) | (0.87-1.31) |  | (2.86) | (0.96-1.50) | **(1.00-1.60)** | (0.98-1.60) | (0.96-1.58) |
|  |  |  | P=0.762 | P=0.580 | P=0.441 | P=0.533 |  |  | P=0.103 | **P=0.049** | P=0.078 | P=0.096 |
| Other specific or moderate learning difficulty | 1237 | 980 | **1.11** | 1.06 | 1.07 | 1.07 |  | 301 | **0.87** | 0.88 | 0.88 | 0.88 |
|  | (7.23) | (8.87) | **(1.02-1.22)** | (0.96-1.18) | (0.96-1.19) | (0.96-1.20) |  | (7.29) | **(0.76-0.99)** | (0.77-1.02) | (0.76-1.02) | (0.76-1.02) |
|  |  |  | **P=0.022** | P=0.251 | P=0.242 | P=0.198 |  |  | **P=0.041** | P=0.082 | P=0.079 | P=0.093 |
| Sensory impairment | 106 | 95 | 1.29 | 1.23 | 1.19 | 1.18 |  | 28 | 0.99 | 0.98 | 0.96 | 0.95 |
|  | (0.62) | (0.86) | (0.97-1.71) | (0.89-1.70) | (0.85-1.68) | (0.83-1.67) |  | (0.68) | (0.63-1.54) | (0.63-1.54) | (0.60-1.53) | (0.60-1.51) |
|  |  |  | P=0.081 | P=0.202 | P=0.315 | P=0.350 |  |  | P=0.964 | P=0.932 | P=0.865 | P=0.832 |
| Physical or motor impairment | 143 | 113 | 1.13 | 1.15 | 1.08 | 1.08 |  | 36 | 0.93 | 0.97 | 0.87 | 0.87 |
|  | (0.84) | (1.02) | (0.88-1.44) | (0.87-1.54) | (0.80-1.47) | (0.79-1.46) |  | (0.87) | (0.65-1.35) | (0.67-1.40) | (0.59-1.29) | (0.59-1.28) |
|  |  |  | P=0.351 | P=0.329 | P=0.620 | P=0.631 |  |  | P=0.714 | P=0.877 | P=0.483 | P=0.476 |
| Language or speech disorder | 619 | 458 | **1.16** | 1.11 | 1.06 | 1.08 |  | 95 | **0.64** | **0.66** | **0.60** | **0.61** |
|  | (3.62) | (4.15) | **(1.02-1.32)** | (0.97-1.28) | (0.91-1.23) | (0.92-1.25) |  | (2.30) | **(0.51-0.80)** | **(0.52-0.82)** | **(0.48-0.77)** | **(0.48-0.77)** |
|  |  |  | **P=0.022** | P=0.140 | P=0.457 | P=0.351 |  |  | **P<0.001** | **P<0.001** | **P<0.001** | **P<0.001** |
| Autistic spectrum disorder | 249 | 155 | 0.94 | 0.96 | 0.97 | 0.96 |  | 78 | 1.26 | 1.24 | 1.21 | 1.21 |
|  | (1.46) | (1.40) | (0.76-1.16) | (0.76-1.21) | (0.76-1.23) | (0.76-1.23) |  | (1.89) | (0.97-1.64) | (0.95-1.64) | (0.91-1.62) | (0.91-1.61) |
|  |  |  | P=0.557 | P=0.738 | P=0.794 | P=0.765 |  |  | P=0.078 | P=0.117 | P=0.192 | P=0.200 |
| Social, emotional & behavioural difficulty or mental health problem | 711 | 571 | **1.15** | 1.03 | 1.07 | 1.08 |  | 189 | 0.98 | 0.96 | 0.95 | 0.96 |
|  | (4.16) | (5.17) | **(1.02-1.29)** | (0.90-1.18) | (0.93-1.23) | (0.94-1.24) |  | (4.58) | (0.83-1.16) | (0.81-1.15) | (0.79-1.15) | (0.80-1.16) |
|  |  |  | **P=0.022** | P=0.637 | P=0.356 | P=0.287 |  |  | P=0.825 | P=0.660 | P=0.623 | P=0.684 |
| Physical health problem | 233 | 158 | 0.98 | 1.08 | 1.1 | 1.11 |  | 64 | 1.04 | 1.12 | 1.17 | 1.18 |
|  | (1.36) | (1.43) | (0.80-1.21) | (0.86-1.36) | (0.86-1.40) | (0.87-1.42) |  | (1.55) | (0.79-1.38) | (0.84-1.49) | (0.86-1.59) | (0.86-1.61) |
|  |  |  | P=0.847 | P=0.513 | P=0.444 | P=0.399 |  |  | P=0.775 | P=0.449 | P=0.320 | P=0.297 |

**caesarean section compared to ERCS at ≥ 39 weeks’ gestation**

^a^Base model adjusted for year of birth only.

^b^Model A adjusted for year of birth, socio-demographic (maternal age, mother's country of birth, marital status, socioeconomic status and child’s ethnicity) and maternal medical and pregnancy-related factors (number of previous caesarean sections, any prior vaginal birth, inter-pregnancy interval, maternal smoking status at booking, maternal BMI at booking, hypertensive disorder, diabetes and prelabour rupture of membranes).

^c^Model B, adjusted for variables in Model A and additionally adjusted for infant-related factors (sex of infant, gestational age at birth and birth weight centile).

^d^Model C, adjusted for variables in Model B and additionally adjusted for any breastfeeding at 6-8 weeks postpartum.

Bold text indicates statistically significant findings at the 5% level.

Abbreviations: BMI, body mass index; CI, confidence interval; ERCS, elective repeat caesarean section; OR, odds ratio; RR, risk ratio; SENs, special educational needs; VBAC, vaginal birth after previous caesarean

**Table S11. Outcomes following planned VBAC compared to ERCS, analysing SEN as a repeated measures yearly outcome***

| **Outcomes** | **Base model^a^ OR or RR (95% CI)** | **Model A^b^ OR or RR (95% CI)** | **Model B^c^ OR or RR (95% CI)** | **Model C^d^ OR or RR (95% CI)** |
| --- | --- | --- | --- | --- |
| Any SENs | 1.03 (0.98-1.09) | 1.03 (0.97-1.08) | 1.04 (0.99-1.10) | 1.05 (0.99-1.11) |
|  | P=0.189 | P=0.331 | P=0.149 | P=0.111 |
| Learning disability | 0.98 (0.86-1.13) | 1.00 (0.87-1.16) | 1.05 (0.90-1.22) | 1.06 (0.91-1.24) |
|  | P=0.822 | P=0.980 | P=0.561 | P=0.471 |
| Dyslexia | 1.08 (0.93-1.27) | 1.16 (0.97-1.38) | 1.15 (0.96-1.38) | 1.14 (0.95-1.37) |
|  | P=0.308 | P=0.097 | P=0.126 | P=0.153 |
| Other specific or moderate learning difficulty | 0.99 (0.91-1.08) | 1.00 (0.92-1.10) | 1.03 (0.93-1.13) | 1.03 (0.94-1.14) |
|  | P=0.861 | P=0.934 | P=0.585 | P=0.485 |
| Sensory impairment | 1.06 (0.82-1.36) | 1.12 (0.85-1.48) | 1.21 (0.90-1.61) | 1.20 (0.90-1.60) |
|  | P=0.674 | P=0.411 | P=0.201 | P=0.214 |
| Physical or motor impairment | 1.02 (0.81-1.28) | 1.08 (0.84-1.38) | 1.17 (0.89-1.52) | 1.16 (0.89-1.52) |
|  | P=0.862 | P=0.559 | P=0.262 | P=0.265 |
| Language or speech disorder | 0.96 (0.85-1.08) | 0.93 (0.81-1.07) | 0.92 (0.80-1.06) | 0.93 (0.80-1.07) |
|  | P=0.494 | P=0.297 | P=0.251 | P=0.301 |
| Autistic spectrum disorder | 1.00 (0.83-1.20) | 0.98 (0.80-1.21) | 1.05 (0.85-1.30) | 1.04 (0.84-1.29) |
|  | P=0.983 | P=0.872 | P=0.655 | P=0.724 |
| Social, emotional and behavioural difficulty or mental health problem | **1.13 (1.01-1.26)** | 1.02 (0.90-1.16) | 1.04 (0.91-1.19) | 1.05 (0.92-1.20) |
|  | **P=0.027** | P=0.734 | P=0.529 | P=0.468 |
| Physical health problem | 1.02 (0.84-1.24) | 1.06 (0.86-1.32) | 1.08 (0.86-1.35) | 1.09 (0.87-1.36) |
|  | P=0.854 | P=0.583 | P=0.503 | P=0.458 |

^a^Base model adjusted for year of birth and age of child at the time of the pupil census.

^b^Model A adjusted for variables in base model and additionally adjusted for, socio-demographic (maternal age, mother's country of birth, marital status, socioeconomic status and child’s ethnicity) and maternal medical and pregnancy-related factors (number of previous caesarean sections, any prior vaginal birth, inter-pregnancy interval, maternal smoking status at booking, maternal BMI at booking, hypertensive disorder, diabetes and prelabour rupture of membranes).

^c^Model B, adjusted for variables in Model A and additionally adjusted for infant-related factors (sex of infant, gestational age at birth and birth weight centile).

^d^Model C, adjusted for variables in Model B and additionally adjusted for any breastfeeding at 6-8 weeks postpartum.

*Median number of pupil census years per child when they were aged 4-11 years and attending a primary or special school was 5 (IQR 3-7).

Bold text indicates statistically significant findings at the 5% level.

Abbreviations: BMI, body mass index; CI, confidence interval; ERCS, elective repeat caesarean section; OR, odds ratio; RR, risk ratio; SENs, special educational needs; VBAC, vaginal birth after previous caesarean

**Table S12. Outcomes following planned VBAC with and without labour induction compared to ERCS, analysing SEN as a repeated measures yearly outcome***

| **Outcomes** | **Planned VBAC without labour induction vs. ERCS** | | | |  | **Planned VBAC with labour induction vs. ERCS** | | | | |
| --- | --- | --- | --- | --- | --- | --- | --- | --- | --- | --- |
|  | **Base model^a^ OR or RR (95% CI)** | **Model A^b^ OR or RR (95% CI)** | **Model B^c^ OR or RR (95% CI)** | **Model C^d^ OR or RR (95% CI)** |  | | **Base model^a^ OR or RR (95% CI)** | **Model A^b^ OR or RR (95% CI)** | **Model B^c^ OR or RR (95% CI)** | **Model C^d^ OR or RR (95% CI)** |
| Any SENs | 1.01 | 1.02 | 1.03 | 1.03 |  | | **1.15** | 1.07 | **1.11** | **1.11** |
|  | (0.96-1.06) | (0.96-1.08) | (0.97-1.09) | (0.98-1.09) |  | | **(1.05-1.26)** | (0.98-1.17) | **(1.01-1.21)** | **(1.01-1.22)** |
|  | P=0.769 | P=0.536 | P=0.327 | P=0.258 |  | | **P=0.002** | P=0.151 | **P=0.032** | **P=0.027** |
| Learning disability | 0.96 | 0.99 | 1.03 | 1.04 |  | | 1.13 | 1.08 | 1.18 | 1.19 |
|  | (0.83-1.11) | (0.85-1.15) | (0.87-1.20) | (0.88-1.22) |  | | (0.89-1.43) | (0.85-1.37) | (0.92-1.53) | (0.92-1.54) |
|  | P=0.542 | P=0.871 | P=0.762 | P=0.659 |  | | P=0.305 | P=0.513 | P=0.196 | P=0.176 |
| Dyslexia | 1.04 | 1.12 | 1.12 | 1.11 |  | | 1.25 | 1.31 | 1.31 | 1.30 |
|  | (0.88-1.23) | (0.93-1.34) | (0.93-1.35) | (0.92-1.33) |  | | (0.95-1.63) | (0.98-1.75) | (0.97-1.76) | (0.96-1.75) |
|  | P=0.628 | P=0.226 | P=0.241 | P=0.287 |  | | P=0.105 | P=0.064 | P=0.077 | P=0.084 |
| Other specific or moderate learning difficulty | 0.99 | 1.01 | 1.03 | 1.04 |  | | 1.03 | 0.97 | 1.01 | 1.02 |
|  | (0.90-1.08) | (0.92-1.12) | (0.94-1.14) | (0.94-1.15) |  | | (0.89-1.20) | (0.83-1.13) | (0.86-1.20) | (0.87-1.20) |
|  | P=0.754 | P=0.759 | P=0.516 | P=0.421 |  | | P=0.657 | P=0.704 | P=0.866 | P=0.814 |
| Sensory impairment | 0.95 | 1.02 | 1.08 | 1.07 |  | | 1.50 | **1.55** | **1.76** | **1.75** |
|  | (0.72-1.25) | (0.75-1.37) | (0.80-1.47) | (0.79-1.46) |  | | (0.99-2.27) | **(1.01-2.38)** | **(1.12-2.76)** | **(1.12-2.74)** |
|  | P=0.697 | P=0.922 | P=0.617 | P=0.647 |  | | P=0.057 | **P=0.044** | **P=0.014** | **P=0.014** |
| Physical or motor impairment | 1.05 | 1.11 | 1.19 | 1.19 |  | | 0.89 | 0.90 | 1.02 | 1.02 |
|  | (0.82-1.33) | (0.86-1.45) | (0.90-1.56) | (0.90-1.56) |  | | (0.58-1.38) | (0.58-1.40) | (0.63-1.63) | (0.63-1.63) |
|  | P=0.717 | P=0.425 | P=0.219 | P=0.222 |  | | P=0.611 | P=0.637 | P=0.945 | P=0.948 |
| Language or speech disorder | 0.94 | 0.93 | 0.92 | 0.93 |  | | 1.03 | 0.91 | 0.91 | 0.92 |
|  | (0.82-1.07) | (0.81-1.08) | (0.79-1.07) | (0.80-1.08) |  | | (0.82-1.31) | (0.71-1.16) | (0.71-1.18) | (0.71-1.18) |
|  | P=0.354 | P=0.339 | P=0.268 | P=0.321 |  | | P=0.795 | P=0.448 | P=0.478 | P=0.507 |
| Autistic spectrum disorder | 0.97 | 0.97 | 1.03 | 1.01 |  | | 1.10 | 1.01 | 1.14 | 1.13 |
|  | (0.80-1.19) | (0.79-1.21) | (0.82-1.28) | (0.81-1.27) |  | | (0.79-1.54) | (0.71-1.43) | (0.80-1.63) | (0.79-1.62) |
|  | P=0.784 | P=0.812 | P=0.827 | P=0.901 |  | | P=0.567 | P=0.970 | P=0.471 | P=0.499 |
| Social, emotional and behavioural difficulty or mental health problem | 1.09 | 1.01 | 1.03 | 1.04 |  | | **1.35** | 1.09 | 1.16 | 1.16 |
|  | (0.97-1.22) | (0.89-1.16) | (0.90-1.18) | (0.90-1.19) |  | | **(1.12-1.64)** | (0.87-1.36) | (0.92-1.45) | (0.93-1.46) |
|  | P=0.164 | P=0.840 | P=0.682 | P=0.613 |  | | **P=0.002** | P=0.447 | P=0.211 | P=0.195 |
| Physical health problem | 0.98 | 1.03 | 1.05 | 1.06 |  | | 1.23 | 1.22 | 1.28 | 1.29 |
|  | (0.79-1.20) | (0.82-1.30) | (0.83-1.32) | (0.83-1.34) |  | | (0.87-1.73) | (0.85-1.75) | (0.88-1.87) | (0.88-1.89) |
|  | P=0.823 | P=0.791 | P=0.704 | P=0.650 |  | | P=0.241 | P=0.274 | P=0.196 | P=0.185 |

^a^Base model adjusted for year of birth and age of child at the time of the pupil census.

^b^Model A adjusted for variables in base model and additionally adjusted for, socio-demographic (maternal age, mother's country of birth, marital status, socioeconomic status and child’s ethnicity) and maternal medical and pregnancy-related factors (number of previous caesarean sections, any prior vaginal birth, inter-pregnancy interval, maternal smoking status at booking, maternal BMI at booking, hypertensive disorder, diabetes and prelabour rupture of membranes).

^c^Model B, adjusted for variables in Model A and additionally adjusted for infant-related factors (sex of infant, gestational age at birth and birth weight centile).

^d^Model C, adjusted for variables in Model B and additionally adjusted for any breastfeeding at 6-8 weeks postpartum.

*Median number of pupil census years per child when they were aged 4-11 years and attending a primary or special school was 5 (IQR 3-7).

Bold text indicates statistically significant findings at the 5% level.

Abbreviations: BMI, body mass index; CI, confidence interval; ERCS, elective repeat caesarean section; OR, odds ratio; RR, risk ratio; SENs, special educational needs; VBAC, vaginal birth after previous caesarean

**Table S13. Outcomes according to actual mode of birth – planned and actually had a VBAC and planned VBAC but had in-labour non-elective repeat**

| **Outcomes** | **Planned and actually had VBAC vs. ERCS** | | | |  | **Planned VBAC but had in-labour non-elective repeat caesarean section vs. ERCS** | | | | |
| --- | --- | --- | --- | --- | --- | --- | --- | --- | --- | --- |
|  | **Base model^a^ OR or RR (95% CI)** | **Model A^b^ OR or RR (95% CI)** | **Model B^c^ OR or RR (95% CI)** | **Model C^d^ OR or RR (95% CI)** |  | | **Base model^a^ OR or RR (95% CI)** | **Model A^b^ OR or RR (95% CI)** | **Model B^c^ OR or RR (95% CI)** | **Model C^d^ OR or RR (95% CI)** |
| Any SENs | 1.05 | 1.03 | 1.05 | 1.06 |  | | 0.99 | 1.01 | 1.02 | 1.02 |
|  | (1.00-1.11) | (0.98-1.10) | (0.99-1.12) | (1.00-1.13) |  | | (0.92-1.07) | (0.94-1.09) | (0.94-1.10) | (0.94-1.10) |
|  | P=0.069 | P=0.254 | P=0.087 | P=0.063 |  | | P=0.847 | P=0.793 | P=0.688 | P=0.621 |
| Learning disability | 1.00 | 1.01 | 1.06 | 1.07 |  | | 0.94 | 0.99 | 1.03 | 1.04 |
|  | (0.86-1.17) | (0.86-1.18) | (0.89-1.25) | (0.90-1.26) |  | | (0.76-1.16) | (0.80-1.22) | (0.82-1.29) | (0.83-1.30) |
|  | P=0.950 | P=0.914 | P=0.528 | P=0.440 |  | | P=0.56 | P=0.91 | P=0.80 | P=0.74 |
| Dyslexia | 1.05 | 1.13 | 1.13 | 1.12 |  | | 1.15 | 1.22 | 1.19 | 1.18 |
|  | (0.89-1.26) | (0.93-1.37) | (0.92-1.38) | (0.92-1.37) |  | | (0.92-1.43) | (0.96-1.54) | (0.94-1.52) | (0.93-1.50) |
|  | P=0.550 | P=0.217 | P=0.231 | P=0.272 |  | | P=0.215 | P=0.102 | P=0.146 | P=0.166 |
| Other specific or moderate learning difficulty | 1.04 | 1.03 | 1.06 | 1.07 |  | | 0.89 | 0.94 | 0.96 | 0.96 |
|  | (0.95-1.14) | (0.94-1.14) | (0.96-1.17) | (0.96-1.18) |  | | (0.79-1.02) | (0.82-1.07) | (0.84-1.10) | (0.84-1.11) |
|  | P=0.429 | P=0.496 | P=0.262 | P=0.205 |  | | P=0.086 | P=0.368 | P=0.542 | P=0.607 |
| Sensory impairment | 1.06 | 1.10 | 1.17 | 1.17 |  | | 1.06 | 1.18 | 1.28 | 1.27 |
|  | (0.80-1.40) | (0.80-1.50) | (0.85-1.62) | (0.85-1.61) |  | | (0.72-1.56) | (0.80-1.75) | (0.86-1.89) | (0.86-1.88) |
|  | P=0.707 | P=0.564 | P=0.329 | P=0.347 |  | | P=0.776 | P=0.405 | P=0.226 | P=0.234 |
| Physical or motor impairment | 1.05 | 1.11 | 1.21 | 1.21 |  | | 0.95 | 1.01 | 1.07 | 1.07 |
|  | (0.82-1.35) | (0.84-1.48) | (0.90-1.63) | (0.90-1.63) |  | | (0.67-1.34) | (0.70-1.44) | (0.74-1.55) | (0.74-1.55) |
|  | P=0.693 | P=0.453 | P=0.199 | P=0.201 |  | | P=0.771 | P=0.974 | P=0.713 | P=0.717 |
| Language or speech disorder | 1.07 | 1.04 | 1.03 | 1.04 |  | | **0.71** | **0.71** | **0.69** | **0.70** |
|  | (0.93-1.22) | (0.90-1.21) | (0.89-1.21) | (0.89-1.22) |  | | **(0.58-0.88)** | **(0.57-0.88)** | **(0.55-0.87)** | **(0.56-0.87)** |
|  | P=0.340 | P=0.601 | P=0.667 | P=0.587 |  | | **P=0.002** | **P=0.002** | **P=0.001** | **P=0.002** |
| Autistic spectrum disorder | 0.91 | 0.90 | 0.98 | 0.97 |  | | 1.20 | 1.14 | 1.19 | 1.18 |
|  | (0.73-1.12) | (0.72-1.14) | (0.77-1.24) | (0.76-1.23) |  | | (0.92-1.57) | (0.86-1.50) | (0.90-1.57) | (0.89-1.56) |
|  | P=0.369 | P=0.397 | P=0.846 | P=0.777 |  | | P=0.169 | P=0.369 | P=0.234 | P=0.259 |
| Social, emotional and behavioural difficulty or mental health problem | **1.19** | 1.06 | 1.09 | 1.10 |  | | 1.00 | 0.93 | 0.93 | 0.93 |
|  | **(1.06-1.34)** | (0.92-1.21) | (0.94-1.26) | (0.95-1.27) |  | | (0.85-1.18) | (0.77-1.14) | (0.76-1.14) | (0.76-1.14) |
|  | **P=0.004** | P=0.444 | P=0.239 | P=0.204 |  | | P=1.000 | P=0.504 | P=0.464 | P=0.501 |
| Physical health problem | 0.98 | 1.03 | 1.04 | 1.04 |  | | 1.11 | 1.13 | 1.17 | 1.18 |
|  | (0.78-1.22) | (0.81-1.31) | (0.81-1.33) | (0.81-1.34) |  | | (0.84-1.47) | (0.84-1.51) | (0.86-1.58) | (0.87-1.60) |
|  | P=0.834 | P=0.820 | P=0.786 | P=0.732 |  | | P=0.458 | P=0.423 | P=0.312 | P=0.291 |

**caesarean section compared to ERCS, analysing SEN as a repeated measures yearly outcome***

^a^Base model adjusted for year of birth and age of child at the time of the pupil census.

^b^Model A adjusted for variables in base model and additionally adjusted for, socio-demographic (maternal age, mother's country of birth, marital status, socioeconomic status and child’s ethnicity) and maternal medical and pregnancy-related factors (number of previous caesarean sections, any prior vaginal birth, inter-pregnancy interval, maternal smoking status at booking, maternal BMI at booking, hypertensive disorder, diabetes and prelabour rupture of membranes).

^c^Model B, adjusted for variables in Model A and additionally adjusted for infant-related factors (sex of infant, gestational age at birth and birth weight centile).

^d^Model C, adjusted for variables in Model B and additionally adjusted for any breastfeeding at 6-8 weeks postpartum.

*Median number of pupil census years per child when they were aged 4-11 years and attending a primary or special school was 5 (IQR 3-7).

Bold text indicates statistically significant findings at the 5% level.

Abbreviations: BMI, body mass index; CI, confidence interval; ERCS, elective repeat caesarean section; OR, odds ratio; RR, risk ratio; SENs, special educational needs; VBAC, vaginal birth after previous caesarean

**Table S14**. **Outcomes following planned VBAC compared to ERCS, removing covariate maternal BMI from the analysis**

| **Outcomes** | **Base model^a^ OR or RR (95% CI)** | **Model A^b^ OR or RR (95% CI)** | **Model B^c^ OR or RR (95% CI)** | **Model C^d^ OR or RR (95% CI)** |
| --- | --- | --- | --- | --- |
| Any SENs | 1.03 (0.99-1.07) | 1.00 (0.96-1.05) | 1.01 (0.97-1.06) | 1.02 (0.98-1.07) |
|  | P=0.194 | P=0.963 | P=0.540 | P=0.385 |
| Learning disability | 1.02 (0.91-1.14) | 0.96 (0.84-1.09) | 0.99 (0.86-1.13) | 1.01 (0.88-1.15) |
|  | P=0.754 | P=0.487 | P=0.868 | P=0.905 |
| Dyslexia | 1.02 (0.89-1.17) | 1.09 (0.94-1.27) | 1.10 (0.94-1.28) | 1.08 (0.93-1.26) |
|  | P=0.765 | P=0.232 | P=0.228 | P=0.310 |
| Other specific or moderate learning difficulty | 1.02 (0.95-1.09) | 0.99 (0.92-1.07) | 1.01 (0.93-1.10) | 1.02 (0.94-1.11) |
|  | P=0.658 | P=0.777 | P=0.771 | P=0.621 |
| Sensory impairment | 1.05 (0.85-1.30) | 1.07 (0.84-1.36) | 1.14 (0.89-1.47) | 1.13 (0.88-1.46) |
|  | P=0.633 | P=0.578 | P=0.286 | P=0.320 |
| Physical or motor impairment | 1.03 (0.86-1.24) | 1.00 (0.81-1.24) | 1.06 (0.85-1.32) | 1.07 (0.86-1.34) |
|  | P=0.731 | P=0.978 | P=0.606 | P=0.549 |
| Language or speech disorder | 1.02 (0.92-1.12) | 0.96 (0.86-1.08) | 0.95 (0.84-1.06) | 0.96 (0.85-1.08) |
|  | P=0.762 | P=0.516 | P=0.365 | P=0.501 |
| Autistic spectrum disorder | 0.97 (0.84-1.13) | 0.94 (0.79-1.11) | 0.96 (0.81-1.14) | 0.96 (0.81-1.15) |
|  | P=0.732 | P=0.443 | P=0.663 | P=0.673 |
| Social, emotional and & behavioural difficulty or mental health problem | 1.12 (1.02-1.22) | 1.01 (0.91-1.12) | 1.03 (0.93-1.14) | 1.04 (0.94-1.16) |
|  | P=0.017 | P=0.860 | P=0.597 | P=0.466 |
| Physical health problem | 0.98 (0.84-1.15) | 1.01 (0.85-1.21) | 1.04 (0.87-1.24) | 1.05 (0.87-1.26) |
|  | P=0.822 | P=0.871 | P=0.698 | P=0.621 |

^a^Base model adjusted for year of birth and age of child at the time of the pupil census.

^b^Model A adjusted for variables in base model and additionally adjusted for, socio-demographic (maternal age, mother's country of birth, marital status, socioeconomic status and child’s ethnicity) and maternal medical and pregnancy-related factors (number of previous caesarean sections, any prior vaginal birth, inter-pregnancy interval, maternal smoking status at booking, hypertensive disorder, diabetes and prelabour rupture of membranes).

^c^Model B, adjusted for variables in Model A and additionally adjusted for infant-related factors (sex of infant, gestational age at birth and birth weight centile).

^d^Model C, adjusted for variables in Model B and additionally adjusted for any breastfeeding at 6-8 weeks postpartum.

Bold text indicates statistically significant findings at the 5% level.

Abbreviations: BMI, body mass index; CI, confidence interval; ERCS, elective repeat caesarean section; OR, odds ratio; RR, risk ratio; SENs, special educational needs; VBAC, vaginal birth after previous caesarean

**Table S15. Outcomes following planned VBAC with and without labour induction compared to ERCS, removing covariate maternal BMI from the analysis**

|  | **Planned VBAC without labour induction vs. ERCS** | | | |  | | **Planned VBAC with labour induction vs. ERCS** | | | | |
| --- | --- | --- | --- | --- | --- | --- | --- | --- | --- | --- | --- |
|  | **Base model^a^ OR or RR (95% CI)** | **Model A^b^ OR or RR (95% CI)** | **Model B^c^ OR or RR (95% CI)** | **Model C^d^ OR or RR (95% CI)** |  | **Base model^a^ OR or RR (95% CI)** | | **Model A^b^ OR or RR (95% CI)** | **Model B^c^ OR or RR (95% CI)** | **Model C^d^ OR or RR (95% CI)** | |
| Any SENs | 1.01 | 0.99 | 1.00 | 1.01 |  | **1.13** | | 1.04 | 1.07 | | **1.08** |
|  | (0.96-1.05) | (0.95-1.04) | (0.96-1.05) | (0.96-1.06) |  | **(1.05-1.21)** | | (0.97-1.12) | (1.00-1.16) | | **(1.00-1.16)** |
|  | P=0.767 | P=0.765 | P=0.865 | P=0.669 |  | **P=0.001** | | P=0.274 | P=0.061 | | **P=0.045** |
| Learning disability | 1.01 | 0.96 | 0.98 | 1.00 |  | 1.09 | | 0.97 | 1.04 | | 1.06 |
|  | (0.89-1.14) | (0.84-1.09) | (0.86-1.13) | (0.87-1.15) |  | (0.89-1.33) | | (0.78-1.20) | (0.83-1.30) | | (0.84-1.32) |
|  | P=0.910 | P=0.521 | P=0.808 | P=0.966 |  | P=0.432 | | P=0.750 | P=0.741 | | P=0.636 |
| Dyslexia | 1.00 | 1.07 | 1.08 | 1.06 |  | 1.11 | | 1.20 | 1.21 | | 1.19 |
|  | (0.86-1.15) | (0.92-1.25) | (0.92-1.26) | (0.90-1.24) |  | (0.88-1.40) | | (0.94-1.53) | (0.93-1.56) | | (0.92-1.54) |
|  | P=0.955 | P=0.403 | P=0.357 | P=0.467 |  | P=0.361 | | P=0.152 | P=0.152 | | P=0.180 |
| Other specific or moderate learning difficulty | 1.00 | 0.98 | 1.01 | 1.01 |  | 1.11 | | 1.02 | 1.06 | | 1.07 |
|  | (0.92-1.07) | (0.91-1.07) | (0.92-1.09) | (0.93-1.10) |  | (0.98-1.27) | | (0.89-1.16) | (0.93-1.22) | | (0.93-1.23) |
|  | P=0.928 | P=0.703 | P=0.906 | P=0.745 |  | P=0.097 | | P=0.821 | P=0.391 | | P=0.338 |
| Sensory impairment | 0.97 | 0.99 | 1.05 | 1.04 |  | 1.38 | | 1.39 | **1.56** | | **1.55** |
|  | (0.77-1.22) | (0.77-1.28) | (0.81-1.37) | (0.80-1.36) |  | (0.97-1.97) | | (0.96-2.02) | **(1.06-2.29)** | | **(1.06-2.27)** |
|  | P=0.794 | P=0.950 | P=0.693 | P=0.745 |  | P=0.072 | | P=0.079 | **P=0.023** | | **P=0.025** |
| Physical or motor impairment | 1.03 | 1.01 | 1.06 | 1.07 |  | 1.01 | | 0.96 | 1.05 | | 1.06 |
|  | (0.85-1.26) | (0.81-1.26) | (0.84-1.33) | (0.85-1.34) |  | (0.71-1.44) | | (0.66-1.38) | (0.71-1.54) | | (0.73-1.55) |
|  | P=0.734 | P=0.940 | P=0.627 | P=0.569 |  | P=0.947 | | P=0.806 | P=0.811 | | P=0.781 |
| Language or speech disorder | 1.01 | 0.97 | 0.96 | 0.97 |  | 1.06 | | 0.92 | 0.91 | | 0.92 |
|  | (0.90-1.12) | (0.87-1.09) | (0.85-1.08) | (0.86-1.09) |  | (0.88-1.28) | | (0.76-1.12) | (0.74-1.12) | | (0.75-1.13) |
|  | P=0.895 | P=0.650 | P=0.464 | P=0.619 |  | P=0.528 | | P=0.427 | P=0.364 | | P=0.426 |
| Autistic spectrum disorder | 0.95 | 0.92 | 0.94 | 0.94 |  | 1.09 | | 1.00 | 1.06 | | 1.06 |
|  | (0.81-1.11) | (0.77-1.10) | (0.79-1.13) | (0.79-1.13) |  | (0.83-1.45) | | (0.74-1.33) | (0.79-1.43) | | (0.79-1.43) |
|  | P=0.519 | P=0.373 | P=0.521 | P=0.528 |  | P=0.522 | | P=0.979 | P=0.709 | | P=0.705 |
| Social, emotional & behavioural difficulty or mental health problem | 1.09 | 1.00 | 1.02 | 1.03 |  | **1.26** | | 1.06 | 1.11 | | 1.13 |
|  | (0.99-1.20) | (0.90-1.11) | (0.91-1.13) | (0.92-1.15) |  | **(1.08-1.48)** | | (0.90-1.25) | (0.94-1.33) | | (0.95-1.34) |
|  | P=0.078 | P=0.955 | P=0.756 | P=0.612 |  | **P=0.004** | | P=0.503 | P=0.221 | | P=0.182 |
| Physical health problem | 0.95 | 0.99 | 1.01 | 1.02 |  | 1.16 | | 1.18 | 1.24 | | 1.25 |
|  | (0.80-1.12) | (0.82-1.19) | (0.83-1.21) | (0.84-1.23) |  | (0.88-1.53) | | (0.88-1.57) | (0.92-1.68) | | (0.93-1.69) |
|  | P=0.526 | P=0.878 | P=0.958 | P=0.874 |  | P=0.290 | | P=0.270 | P=0.158 | | P=0.143 |

^a^Base model adjusted for year of birth and age of child at the time of the pupil census.

^b^Model A adjusted for variables in base model and additionally adjusted for, socio-demographic (maternal age, mother's country of birth, marital status, socioeconomic status and child’s ethnicity) and maternal medical and pregnancy-related factors (number of previous caesarean sections, any prior vaginal birth, inter-pregnancy interval, maternal smoking status at booking, hypertensive disorder, diabetes and prelabour rupture of membranes).

^c^Model B, adjusted for variables in Model A and additionally adjusted for infant-related factors (sex of infant, gestational age at birth and birth weight centile).

^d^Model C, adjusted for variables in Model B and additionally adjusted for any breastfeeding at 6-8 weeks postpartum.

Bold text indicates statistically significant findings at the 5% level.

Abbreviations: BMI, body mass index; CI, confidence interval; ERCS, elective repeat caesarean section; OR, odds ratio; RR, risk ratio; SENs, special educational needs; VBAC, vaginal birth after previous caesarean

**Table S16. Outcomes according to actual mode of birth – planned and actually had a VBAC and planned VBAC but had in-labour non-elective repeat caesarean section compared to ERCS, removing covariate maternal BMI from the analysis**

|  | **Planned and actually had VBAC vs. ERCS** | | | |  | | **Planned VBAC but had in-labour non-elective repeat caesarean section vs. ERCS** | | | | |
| --- | --- | --- | --- | --- | --- | --- | --- | --- | --- | --- | --- |
|  | **Base model^a^ OR or RR (95% CI)** | **Model A^b^ OR or RR (95% CI)** | **Model B^c^ OR or RR (95% CI)** | **Model C^d^ OR or RR (95% CI)** | |  | | **Base model^a^ OR or RR (95% CI)** | **Model A^b^ OR or RR (95% CI)** | **Model B^c^ OR or RR (95% CI)** | **Model C^d^ OR or RR (95% CI)** |
| Any SENs | 1.05 | 1.01 | 1.03 | 1.04 | |  | | 0.96 | 0.97 | 0.98 | 0.98 |
|  | (1.01-1.10) | (0.97-1.06) | (0.98-1.08) | (0.99-1.09) | |  | | (0.91-1.03) | (0.91-1.04) | (0.92-1.04) | (0.92-1.05) |
|  | P=0.026 | P=0.597 | P=0.225 | P=0.142 | |  | | P=0.249 | P=0.418 | P=0.481 | P=0.575 |
| Learning disability | 1.05 | 0.96 | 0.99 | 1.02 | |  | | 0.94 | 0.94 | 0.97 | 0.99 |
|  | (0.93-1.19) | (0.84-1.11) | (0.86-1.15) | (0.88-1.18) | |  | | (0.79-1.13) | (0.78-1.14) | (0.80-1.18) | (0.82-1.20) |
|  | P=0.462 | P=0.583 | P=0.944 | P=0.827 | |  | | P=0.535 | P=0.537 | P=0.798 | P=0.921 |
| Dyslexia | 0.98 | 1.05 | 1.06 | 1.04 | |  | | 1.12 | 1.19 | 1.18 | 1.16 |
|  | (0.84-1.14) | (0.89-1.23) | (0.89-1.26) | (0.88-1.24) | |  | | (0.93-1.36) | (0.98-1.46) | (0.96-1.45) | (0.95-1.43) |
|  | P=0.769 | P=0.597 | P=0.499 | P=0.625 | |  | | P=0.234 | P=0.082 | P=0.122 | P=0.153 |
| Other specific or moderate learning difficulty | 1.07 | 1.02 | 1.05 | 1.06 | |  | | **0.89** | 0.92 | 0.93 | 0.94 |
|  | (0.99-1.15) | (0.94-1.11) | (0.96-1.15) | (0.97-1.16) | |  | | **(0.80-0.99)** | (0.82-1.03) | (0.83-1.05) | (0.83-1.06) |
|  | P=0.101 | P=0.639 | P=0.292 | P=0.209 | |  | | **P=0.038** | P=0.150 | P=0.247 | P=0.298 |
| Sensory impairment | 1.08 | 1.08 | 1.15 | 1.14 | |  | | 0.99 | 1.06 | 1.13 | 1.13 |
|  | (0.85-1.36) | (0.82-1.41) | (0.87-1.52) | (0.86-1.50) | |  | | (0.70-1.39) | (0.75-1.49) | (0.80-1.61) | (0.79-1.60) |
|  | P=0.521 | P=0.589 | P=0.320 | P=0.357 | |  | | P=0.943 | P=0.757 | P=0.487 | P=0.511 |
| Physical or motor impairment | 1.04 | 1.01 | 1.07 | 1.08 | |  | | 1.02 | 0.99 | 1.03 | 1.04 |
|  | (0.85-1.27) | (0.80-1.28) | (0.84-1.37) | (0.85-1.39) | |  | | (0.76-1.36) | (0.74-1.33) | (0.76-1.40) | (0.77-1.41) |
|  | P=0.711 | P=0.943 | P=0.573 | P=0.516 | |  | | P=0.909 | P=0.949 | P=0.839 | P=0.803 |
| Language or speech disorder | **1.12** | 1.06 | 1.05 | 1.07 | |  | | **0.74** | **0.74** | **0.71** | **0.72** |
|  | **(1.01-1.25)** | (0.94-1.20) | (0.93-1.20) | (0.94-1.22) | |  | | **(0.62-0.88)** | **(0.62-0.88)** | **(0.59-0.86)** | **(0.60-0.87)** |
|  | **P=0.034** | P=0.317 | P=0.411 | P=0.290 | |  | | **P=0.001** | **P=0.001** | **P<0.001** | **P=0.001** |
| Autistic spectrum disorder | 0.89 | 0.85 | 0.88 | 0.88 | |  | | 1.19 | 1.13 | 1.13 | 1.13 |
|  | (0.75-1.06) | (0.70-1.03) | (0.73-1.08) | (0.73-1.08) | |  | | (0.96-1.49) | (0.90-1.42) | (0.89-1.42) | (0.89-1.42) |
|  | P=0.178 | P=0.093 | P=0.219 | P=0.223 | |  | | P=0.111 | P=0.291 | P=0.315 | P=0.313 |
| Social, emotional & behavioural difficulty or mental health problem | **1.17** | 1.03 | 1.06 | 1.08 | |  | | 0.98 | 0.96 | 0.95 | 0.96 |
|  | **(1.06-1.29)** | (0.92-1.15) | (0.95-1.19) | (0.96-1.21) | |  | | (0.85-1.13) | (0.83-1.12) | (0.82-1.11) | (0.83-1.12) |
|  | **P=0.002** | P=0.609 | P=0.300 | P=0.217 | |  | | P=0.778 | P=0.615 | P=0.545 | P=0.622 |
| Physical health problem | 0.95 | 0.98 | 0.99 | 1.01 | |  | | 1.07 | 1.09 | 1.13 | 1.14 |
|  | (0.79-1.13) | (0.80-1.19) | (0.81-1.22) | (0.82-1.23) | |  | | (0.85-1.35) | (0.85-1.39) | (0.88-1.44) | (0.89-1.46) |
|  | P=0.545 | P=0.840 | P=0.959 | P=0.958 | |  | | P=0.567 | P=0.487 | P=0.342 | P=0.312 |

^a^Base model adjusted for year of birth and age of child at the time of the pupil census.

^b^Model A adjusted for variables in base model and additionally adjusted for, socio-demographic (maternal age, mother's country of birth, marital status, socioeconomic status and child’s ethnicity) and maternal medical and pregnancy-related factors (number of previous caesarean sections, any prior vaginal birth, inter-pregnancy interval, maternal smoking status at booking, hypertensive disorder, diabetes and prelabour rupture of membranes).

^c^Model B, adjusted for variables in Model A and additionally adjusted for infant-related factors (sex of infant, gestational age at birth and birth weight centile).

^d^Model C, adjusted for variables in Model B and additionally adjusted for any breastfeeding at 6-8 weeks postpartum.

Bold text indicates statistically significant findings at the 5% level.

Abbreviations: BMI, body mass index; CI, confidence interval; ERCS, elective repeat caesarean section; OR, odds ratio; RR, risk ratio; SENs, special educational needs; VBAC, vaginal birth after previous caesarean

**References**

1. National Records of Scotland. Quality of Data Obtained from the Registration of Births, Stillbirths, Marriages, Civil Partnerships and Deaths <https://www.nrscotland.gov.uk/files//statistics/vital-events/quality-data-obtained-from-registration-of-ve.pdf>. Accessed Accessed 16 May 2018.

2. Information Services Division Scotland. *Births in Scottish Hospitals Technical Report Publication date - 28 November 2017.* Edinburgh2017.

3. Information Services Division Scotland. *Data Quality Assurance Assessment of Maternity Data (SMR02) 2008-2009* Edinburgh2010.

4. Information Services Division Scotland. *Assessment of SMR02 (Maternity Inpatient and Day Case) Data Scotland 2017-2018.* Edinburgh2019.

5. Information Services Division Scotland. *Assessment of SMR01 Data 2010 – 2011.* Edinburgh2012.

6. Information Services Division Scotland. *Infant Feeding Statistics Scotland Publication date - 31 October 2017.* Edinburgh2017.

7. The Scottish Government. *Independent School Census, September 2009.* 2010.

8. The Scottish Government. *Educational Analytical Services Data Quality Process.*

9. Wood R, Clark D, King A, Mackay D, Pell J. Novel cross-sectoral linkage of routine health and education data at an all-Scotland level: a feasibility study. *Lancet.* 2013;382(Sup 3), S10.

10. Scottish Government. School/Pupil Census: Data Specification 2021 uplift. <https://www.gov.scot/publications/scottish-exchange-of-data-school-pupil-census/>.

11. Knight M, Kurinczuk JJ, Spark P, Brocklehurst P. Extreme obesity in pregnancy in the United Kingdom. *Obstet Gynecol.* 2010;115(5):989-997.

12. Information Services Division Scotland. Data dictionary SMR02 Maternity Inpatient and Day Case. <https://www.ndc.scot.nhs.uk/Data-Dictionary/SMR-Datasets/SMR02-Maternity-Inpatient-and-Day-Case/>.

13. Bonellie S, Chalmers J, Gray R, Greer I, Jarvis S, Williams C. Centile charts for birthweight for gestational age for Scottish singleton births. *BMC Pregnancy Childbirth.* 2008;8:5.

14. Royal College of Obstetricians and Gynaecologists. *Birth After Previous Caesarean Birth, Green-top Guideline No. 45* London;2015.

15. National Institute for Health and Clinical Excellence. *Caesarean section NICE clinical guideline 132,.* 2011.

16. van den Berg A, van Elburg RM, van Geijn HP, Fetter WP. Neonatal respiratory morbidity following elective caesarean section in term infants. A 5-year retrospective study and a review of the literature. *Eur J Obstet Gynecol Reprod Biol.* 2001;98(1):9-13.

17. Hansen AK, Wisborg K, Uldbjerg N, Henriksen TB. Elective caesarean section and respiratory morbidity in the term and near-term neonate. *Acta Obstet Gynecol Scand.* 2007;86(4):389-394.

18. Boutsikou T, Malamitsi-Puchner A. Caesarean section: impact on mother and child. *Acta Paediatr.* 2011;100(12):1518-1522.

19. MacKay DF, Smith GC, Dobbie R, Pell JP. Gestational age at delivery and special educational need: retrospective cohort study of 407,503 schoolchildren. *PLoS Med.* 2010;7(6):e1000289.

20. Paranjothy S, Dunstan F, Watkins WJ, et al. Gestational age, birth weight, and risk of respiratory hospital admission in childhood. *Pediatrics.* 2013;132(6):e1562-1569.

21. Lutsiv O, Giglia L, Pullenayegum E, et al. A population-based cohort study of breastfeeding according to gestational age at term delivery. *J Pediatr.* 2013;163(5):1283-1288.
